# Quality of Private and Public Ambulatory Health Care in Low and Middle Income Countries: Systematic Review of Comparative Studies

**DOI:** 10.1371/journal.pmed.1000433

**Published:** 2011-04-12

**Authors:** Sima Berendes, Peter Heywood, Sandy Oliver, Paul Garner

**Affiliations:** 1 Liverpool School of Tropical Medicine, Liverpool, United Kingdom; 2 Menzies Center for Health Policy, University of Sydney, Sydney, Australia; 3 Institute of Education, University of London, London, United Kingdom; King's College London, United Kingdom

## Abstract

**Background:**

In developing countries, the private sector provides a substantial proportion of primary health care to low income groups for communicable and non-communicable diseases. These providers are therefore central to improving health outcomes. We need to know how their services compare to those of the public sector to inform policy options.

**Methods and Findings:**

We summarised reliable research comparing the quality of formal private versus public ambulatory health care in low and middle income countries. We selected studies against inclusion criteria following a comprehensive search, yielding 80 studies. We compared quality under standard categories, converted values to a linear 100% scale, calculated differences between providers within studies, and summarised median values of the differences across studies. As the results for for-profit and not-for-profit providers were similar, we combined them. Overall, median values indicated that many services, irrespective of whether public or private, scored low on infrastructure, clinical competence, and practice. Overall, the private sector performed better in relation to drug supply, responsiveness, and effort. No difference between provider groups was detected for patient satisfaction or competence. Synthesis of qualitative components indicates the private sector is more client centred.

**Conclusions:**

Although data are limited, quality in both provider groups seems poor, with the private sector performing better in drug availability and aspects of delivery of care, including responsiveness and effort, and possibly being more client orientated. Strategies seeking to influence quality in both groups are needed to improve care delivery and outcomes for the poor, including managing the increasing burden of non-communicable diseases.

*Please see later in the article for the Editors' Summary*

## Abstract 2

Paul Garner and colleagues conducted a systematic review of 80 studies to compare the quality of private versus public ambulatory health care in low- and middle-income countries.

## Editors' Summary

**Background:**

The provision of private (“for-profit” hospitals and self-employed practitioners, and “not-for-profit” non-government providers, including faith-based organizations) versus public health care services in low and middle income countries raises considerable ideological debate. Ideological arguments aside—which can be very passionate on both sides—there is general agreement that improving the quality of both public and private health care could have a major impact on improved health outcomes, especially as the private sector is so widely used in low and middle income countries. For example, almost three quarters and half of children from the poorest households of South Asia and sub-Saharan Africa, respectively, seek health care from a private provider when they are ill. Private providers are also increasingly responsible for outpatient care for non-communicable diseases.

As a result of the mixed health care system in many low and middle income countries, adequate oversight and stewardship of the mixed system from the national government is essential yet often missing.

**Why Was This Study Done?:**

An understanding of how quality and performance in the private sector compares with that in the public sector would help governments to prioritize where they need to concentrate their efforts. So, for example, if the private sector is generally providing poorer quality care than the public sector, then there is an imperative to improve the quality and outcomes; on the other hand, if the quality of care offered by the private sector is good, the policy priority is to influence the market to further improve access to such health care for low income groups.

In order to help with this comparison, the researchers wanted to systematically identify and summarize the results of studies that directly compared the quality of care offered by public providers with the one offered by “formal” private providers (recognized by law) and “informal” private providers (providers that are not legally recognized, such as lay health workers and shop keepers). For the purposes of this study the researchers focused their comparison on the private and public provision of outpatient care in low and middle income countries.

**What Did the Researchers Do and Find?:**

In their literature review, the researchers searched for relevant studies reported in English, French, or German and published between January 1970 and April 2009. Only studies that compared private and public outpatient medical services in the same country, at the same time, using the same methods, and which met particular quality criteria, were included in the analysis. The researchers also had strict criteria for including qualitative studies, and they retrieved the full text of articles, contacted study authors where appropriate, and verified with a second researcher most (80%) of the extracted study data. In order to evaluate and compare the studies, the researchers converted study values to a linear 100% scale, calculated differences between providers within studies, and summarized the median values of the differences across studies.

The researchers identified a total of 8,812 relevant titles and abstracts and found 80 studies that included direct quantitative comparisons of public and private formal providers. Ten studies included qualitative data. Most studies were conducted after 1990, and mainly in sub-Saharan Africa (*n* = 39) and Asia and the Pacific (*n* = 23). Most studies did not report socio-economic status of public and private service users, and only five studies presented data by different income groups. No study compared the same individual providers working in public and private care settings. Only two studies compared public providers and private informal providers, so the authors excluded these from subsequent analysis.

For the formal sector, since the results for “for-profit” and “not-for-profit” providers were similar, the researchers decided to combine the results. Overall, the researchers found that the median values indicated that many services, irrespective of whether public or private, scored low (less than 50%) on infrastructure, clinical competence, and practice. Generally, the private sector performed better in relation to drug supply, responsiveness, and effort, but there was no detectable difference between provider groups for patient satisfaction. Furthermore, a synthesis of qualitative data suggested that the private sector may be more client-centered.

**What Do These Findings Mean?:**

Based on the findings of this review, there is a clear need to consider the quality of primary health services in both the public and private sector in order to improve health outcomes in low and middle income countries. These findings also indicate that, for some aspects of care, on average the private sector provided better quality services. The overall low quality of care in both the formal private and public sector found in this review is worrying, and calls for the governments of low and middle income countries to find and implement effective strategies to improve the quality in both sectors. This is particularly important given the increasing volume of conditions that require relatively sophisticated, long-term ambulatory medical care, such as non-communicable diseases.

**Additional Information:**

Please access these Web sites via the online version of this summary at http://dx.doi.org/10.1371/journal.pmed.1000433.

- This study is further discussed in a *PLoS Medicine* Perspective by Jishnu Das
- WHO has more information on health service delivery in low- and middle-income countries
- WHO has more information on noncommunicable diseases
- The World Bank's World Development Report for 2004 addresses health care for poor people

## Introduction

The private sector is the main provider of primary health care for the poor in many low and middle income countries (LMICs). For example, in South Asia about three quarters of children from the poorest income quintile with acute respiratory conditions seeking health care go to a private provider [1], and about 45% of sick children from the poorest income quintile across 26 African countries go to a formal or informal private provider rather than a public provider for health care [2]. Private providers are also increasingly important for providing ambulatory care as non-communicable diseases (NCDs) increase [3].

Private providers may be “formal”, i.e. recognised by law or by legally recognised regulatory authorities, or “informal”, i.e. not recognised [4]. Formal private providers include “for-profit” hospitals and self-employed practitioners, and “not-for-profit” non-governmental organizations (NGOs). NGOs include churches, and are particularly common in Africa, although the for-profit/not-for-profit dichotomy is not so clear cut in practice, with some NGOs simply representing private practitioners securing tax breaks [5],[6]. Informal allopathic providers include “quacks”, lay health workers, drug sellers, and ordinary shop keepers [7].

Advocating that formal for-profit private services are preferable to government provision raises considerable ideological debates [8]–[10]; equally, not-for-profit private providers such as those run by churches are seen by some as good and as providing value for money [11]. Whatever the debates, there is agreement that influencing the quality of both public and private providers could have a major impact on health outcomes. Adequate state stewardship and oversight of these mixed systems is widely advocated [9],[12], but the mechanisms to assure quality are not simple and are of unclear effectiveness [13],[14]. Improving stewardship and oversight is complex, involving resources, management, legislation, and approaches to influence the market [15],[16]. Thus, an understanding of how quality and performance in the formal private sector compares with that of the public sector would help governments to focus strategies to improve delivery. Putting this simply, if the private sector is generally providing poorer quality care than the public sector, then there is an imperative to improve the quality and outcomes; on the other hand, if the quality of private-sector care is good, the priority for policy is to influence the market somehow to further improve access for low income groups.

“Quality” has many dimensions [17], including structural quality, aspects of delivery, and the technical or professional content of care, all of which are likely to influence service use. Each dimension will have complex effects on patient satisfaction, patient use of the service, and outcomes for their health. In addition, each is interrelated: population health outcomes will depend on service use, technical quality, and drug availability, for example. A recent substantive analysis that examined the use of medicines in primary care reported poor quality prescribing for both sectors, with little change over time [18]. The authors also reported the relatively poor quality of data and the need for research assessing the difference between the public and private sector. Thus, our objective was to systematically identify and summarise the results of studies that directly compare the quality of private providers and public services in relation to ambulatory health care in LMICs.

## Methods

### Criteria for Inclusion

We included field-based studies that directly compared service quality in ambulatory care from private versus public medical health services. The purpose was to include studies using the same methods to measure the differences, and in the same countries, to avoid confounding factors related to overall differences in service quality between countries. We included studies conducted in LMICs that assessed ambulatory care, defined as the “delivery of personal health care services on an outpatient basis” [19]. We only included studies that compared private and public services in the same country, at the same time, using the same methods, and which met particular quality criteria (Table S1). “Private” refers to “all organizations and individuals working outside the direct control of the state” [20], and we included only those working within the allopathic medical systems. “Private for-profit providers” included individuals or groups of practitioners in privately owned clinics, hospitals, and pharmacies that operate on a for-profit basis, while “private not-for-profit providers” included practitioners in facilities that operate on a non-profit basis, such as various (missionary or non-missionary) NGOs and private voluntary organizations. Informal providers included those without formal health professional qualifications, such as street vendors and shop keepers. We included studies reported in English, French, or German and published from January 1970 to April 2009. We screened all titles/abstracts found by the search methods described below for potential inclusion, and then carefully applied the detailed inclusion criteria (Table S1) to the full text of those identified in the screening search. Studies using qualitative methods were identified and were included if they (a) used internationally accepted data collection methods (e.g., in-depth interviews, focus group discussion, or observation), (b) indicated the methods used in analysis (e.g., thematic analysis, content analysis, or grounded theory), and (c) presented data by theme or in the form of verbatim quotes.

### Search Methods

The search strategy for Medline can be found in Table S2, and a list of the databases searched in Table S3. In addition, we searched all records of the World Health Organization's (WHO's) library database, WHOLIS (on 27 April 2009), all Service Availability Mapping reports published on the WHO Web site (http://www.who.int/healthinfo/systems/samdocs/en/index.html) (on 5 December 2010) [21], all Service Provision Assessment Survey reports published on the Measure DHS Web site (http://www.measuredhs.com/aboutsurveys/search/search_survey_main.cfm?SrvyTp=type&listtypes=3) (on 3 December 2010) [22], and all research studies published on the Core group Web site (http://www.coregroup.org/) (on 6 December 2010), and we examined reference lists of relevant reviews [23]–[25] and of the included studies.

The search strategies included indexed and free-text terms: health sector, health care, delivery of health care, primary health care, medical care, health clinic, outpatient service, ambulatory care, practitioner, health provider, health provision, hospital, pharmacy, drug vendor, drug seller, drug store, public sector, public, private sector, private, quality of health care, Africa, Asia, South America, developing countries, less developed countries, third world countries, underdeveloped country, low income country, low income nation, middle income country, middle income nation, low and middle income countries.

### Data Collection and Analysis

We applied the inclusion criteria to all titles and abstracts. We retrieved full-text copies of potentially relevant records, and discussed each to resolve uncertainties. We then appraised potential studies against a set of basic minimum methodological criteria to exclude studies where data were unlikely to be reliable (Table S1).

We adapted Donabedian's [17] classification of quality of care using structural, delivery, and technical categories (Table 1). We incorporated “responsiveness” [26] to reflect aspects such as waiting time, communication quality, and dignity, as well as an assessment of the “effort” providers make, such as whether they examine the patient, and the length of the consultation time [27],[28], and we divided technical quality into measures of competence and clinical practice (Table 1).

**Table 1:** Quality categories, sub-categories, and indicators used.

Quality Category	Sub-Category	Description and Indicators
Structural	Building, equipment, materials	Availability and condition of health facilities, and of defined equipment, materials, and supplies
	Drug availability	Availability of essential drugs in health facilities and pharmacies
Delivery	Responsiveness	Waiting time, privacy, confidentiality, staff friendliness, communication, dignity
	Effort	Length of consultation time, whether a physical examination is performed, number of explanations given
	Patient satisfaction	Patients' satisfaction with last consultation
Technical	Competence	Professional knowledge and skills
	Clinical practice	Presence or absence of critical elements of care, whether practice is according to standards or guidelines, proxies for correct prescribing behaviour

S. B. extracted data using a standard form, entered into an Access database, with about 80% verified by a second author to ensure standardisation of coding. We contacted 33 authors for further information, and all but nine authors responded. Standard data describing the study were extracted. If a study reported several comparisons, we selected groups that were most similar within the health system (e.g., public hospitals versus private hospitals, or public health centres versus private clinics). If results were presented separately for different cadres or levels of staff qualification, we chose the comparison group with the staff qualification levels that were most comparable and most frequented by the population. If the latter could not be established, we chose the highest qualified comparison group.

We then separately computed summary measures of (a) the overall level of quality of care in the private and in the public sector and (b) the difference of quality of care between both sectors stratified by quality categories and components. If there were several data measures for one component in a study, we computed the median for all reported measures to calculate a single measure for component quality for the provider. For example, in the case of a public-sector score (on a linear scale, with 100% being the maximum obtainable) of 45% for physical infrastructure, 50% for availability of basic diagnostic equipment, and 60% for availability of basic material, the median for the structural component “building, equipment, and material” would be 50%. The median was also computed for the quality score difference between private and public provider. For example, in case of a difference of +5% in physical infrastructure, +11% in availability of basic diagnostic equipment, and +14% in basic material, the median difference would be +11% for the given comparison in a study. After computing the medians for the overall quality of care and for the difference of care for each single comparison in each study, we computed medians and inter-quartile ranges (IQRs) across all comparisons. The size of the difference and the IQRs of the difference were used to judge whether a difference was evident.

## Results

**Figure 1:**
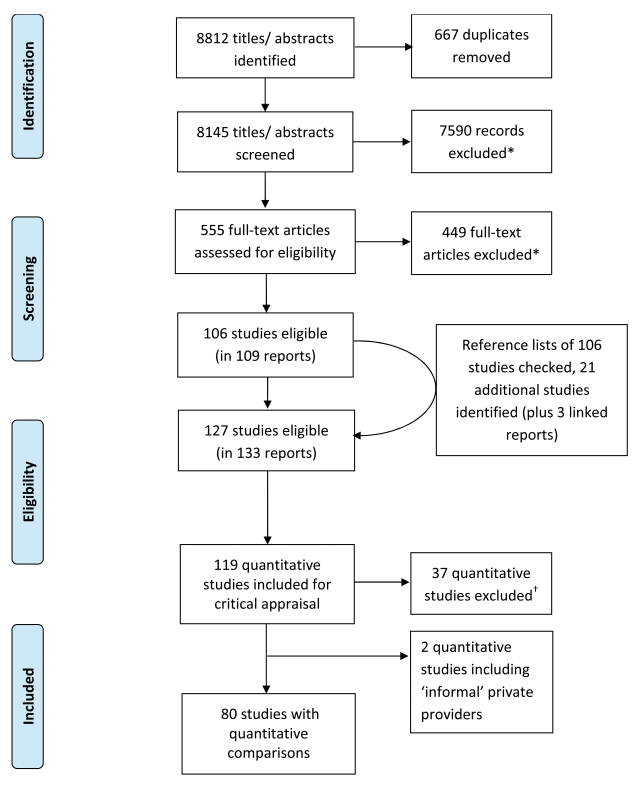
Selection of studies. * See Table S4 for reasons of exclusion; † see Table S5 for reasons of exclusion.

Of 8,812 titles and abstracts identified, 80 studies included direct quantitative comparisons of public and private formal providers (Figure 1, adapted from PRISMA 2009 flow diagram [29]; Tables S4 and S5 describe excluded studies). These yielded 133 comparisons, of which we were able to convert 101 to a 100% scale (Table S6). Most studies were carried out after 1990; they were mainly conducted in sub-Saharan Africa (*n = *39) and in Asia and the Pacific (*n = *23); and most were intended to compare quality, examining all types of primary service and disease category (Table 2; details in Table S9). Most studies did not report socio-economic status of public and private service users, and only five presented data by different wealth groups [30]–[34]. No study compared the same individual providers working in public and private care settings. For two studies [35],[36] that reported results separately for different cadres, we chose public versus private doctors rather than public versus private nurses or midwives as comparison groups, but it should be noted that for both groups results pointed in the same direction.

**Table 2:** Characteristics of quantitative studies comparing public and formal private providers by region (*n = *80).

Characteristic	South Asia, East Asia, and Pacific	Sub-Saharan Africa	Other^a^	Total Number of Studies
**Language**				
English	23	33	16	72
French	0	6	2	8
**Study year range**				
1980–1989	1	2	1	4
1990–1999	8	16	7	31
2000–2009	14	21	10	45
**Primary study purpose**				
Describe or compare quality of private and public services	17	28	13	58
Assess drug availability and affordability	4	3	2	9
Assess demand for, access to, or utilisation of services, or efficiency of service delivery	2	8	3	13
**Service type**				
Promotive or preventive	1	4	2	7
Curative, rehabilitative, or palliative	7	14	7	28
All types	12	18	8	38
Not specified	3	3	1	7
**Disease category**				
Both CD and NCD	14	24	9	47
CD	7	13	5	25
NCD	1	0	3	4
Not specified	1	2	1	4
**Population age**				
Adult	6	11	2	19
Both adult and child	15	21	7	43
Child	1	3	4	8
Not specified	1	5	5	11
**Population gender**				
Both (male and female)	21	34	15	70
Female	2	5	3	10
**Total number of studies**	**23**	**39**	**18**	**80**

a Includes Europe and Central Asia (*n = *1), Latin America and the Caribbean (*n = *6), the Middle East and North Africa (*n = *7), and studies reporting on countries in more than one world region (*n = *4).

We found only two studies comparing public providers and private informal providers. The first [37] compared malaria-related knowledge and chloroquine availability in public dispensaries and informal drug vendors, and suggested that the public sector was slightly better. The second [38] mixed both formal and informal private providers together. These two studies were excluded from further analysis.

Of the 101 formal private versus public sector comparisons that were converted to a 100% scale, 57 compared government with private for-profit providers, 10 with a mix of for-profit and not-for-profit providers, and 34 with private not-for-profit providers. Of the last 34 comparisons, most (*n = *29) were conducted in sub-Saharan Africa.

Study-level summary values for each quality component are presented in Table 3, along with the summary of the within-study differences. We also carried out an analysis that separated private for-profit and private not-for-profit providers (Table S7). As the results in the for-profit and not-for-profit providers were remarkably consistent, they are presented as combined.

**Table 3:** Overall level of quality and comparative quality difference of public and formal private providers.

Category	Component	Number of Comparisons Converted to 100% Scale	Public Quality Score (%)		Private Quality Score (%)		Difference Private-Public^a^ (%)	
			Median	IQR	Median	IQR	Median	IQR
Structural	Building, equipment, and materials	26	41.9	25.0, 76.5	44.5	22.0, 86.6	2.8	−2.9, 20.6
	Drug availability	14	45.3	38.8, 58.5	63.0	45.4, 94.8	17.9	12.5, 29.1
Delivery	Responsiveness	7	85.0	56.9, 86.3	89.1	75.7, 94.5	7.5	7.0,12.4
	Effort	3	84.9	46.5, 87.0	92.9	54.5, 93.5	8.0	5.5, 8.0
	Patient satisfaction	10	75.0	56.9, 78.8	75.0	68.0, 79.1	0.5	−2.0, 4.4
Technical	Competence	19	52.8	36.3, 54.2	45.2	35.0, 53.3	−3.0	−7.6, 0.8
	Clinical practice	22	44.5	27.5, 60.9	47.0	39.1, 66.5	5.2	1.3, 14.0

a Within each comparison, the difference between the public score and the private score was calculated. The data in this column are the median of these values across all studies. For this reason, they will not correspond to an arithmetic difference of the absolute median scores in the previous columns.

In addition, ten studies included qualitative data that met our eligibility criteria, with a similar geographic spread to the quantitative data.

### Structure

For buildings, equipment, materials, and supplies, no difference was detected. For the 26 comparisons, the IQR of the difference included 0. Respondents in two qualitative studies reporting on this category described private facilities as better [39],[40].

For drug availability, private-sector care was substantially better than public-sector care, from 14 comparisons. Nine studies used a standard method and referred to the WHO essential drug list [41],[42]. None of the quantitative studies compared the quality of drugs available in the public versus private sector. Qualitative studies reported that the private sector was more trusted for drug quality [43] and that the drugs were more readily available [39],[40],[44],[45].

### Service Delivery

For responsiveness, private-sector care was better (see Table 1 for definition), from seven comparisons. Studies used patient interviews, observations, or simulated visits. In six of the seven comparisons measuring waiting time, the time was shorter in the private sector. Qualitative data in five studies indicated that the private sector provided more personalised, respectful [39],[40],[46],[47], listening [43], and client-centred service, as well as service that was more convenient [48] and quicker and easier to access [47],[49].

For effort, private-sector care was better, from three comparisons. A further four studies reported on average consultation times, which were longer in the private sector in all studies, although statistical significance was only computed and confirmed in two of them [6],[50]–[52]. Qualitative data were consistent with this finding. Studies consistently reported criticisms of the public sector (with providers showing favouritism for some patients and less respect for poorer clients [39],[40],[43],[44],[46],[48],[49]) and praise for the private sector [39],[40],[43],[48],[49].

For patient satisfaction, no difference between private and public sector was detected, from ten comparisons. None of the studies measuring “satisfaction” reported the use of a validated questionnaire. Only one took into account possible differences in expectations of public and private services [53].

### Technical Quality

For competence, scores for private- versus public-sector care were similar, and generally poor, from 19 comparisons; competence was measured by case scenarios or vignettes, provider interviews, or a formal test. In qualitative studies the private sector was reported as quicker and easier to access, although the competence of some providers was questioned [40],[48]. The public sector was often perceived as technically competent but inconvenient and provider centred, with complex systems that took time and effort to negotiate [44],[47],[49],[54].

For clinical practice, private-sector care was marginally better, from 22 comparisons. Of those not convertible to a linear 100% scale, 14 studies used the same standard methods to assess prescribing behaviour, summarised in Table S8, with no obvious differences. In qualitative studies, respondents perceived public providers as qualified and well trained [43], although some were thought to overprescribe to raise their income [40],[48]. The private sector was also criticised for overprescribing and collusion between doctors and pharmacists [46], for suspected “fake” or unlabelled drugs, for “fake” doctors, and for nurses practicing illegally in private pharmacies in need of regulation [40],[46],[48].

We carried out a sensitivity analysis including only studies and comparisons (*n = *67) classified as high quality because of their size (Table S1 provides the criteria); the results obtained were very similar to Table 3.

### For-Profit and Not-for-Profit Providers

As mentioned above, most of the not-for-profit studies were carried out in sub-Saharan Africa (29 of 34 comparisons). Table S7 contains an analysis stratified by private for-profit and private not-for-profit. The direction of the difference is the same as for the aggregated value for all components. Notably, clinical practice was much better in the for-profit sector, and the difference was less marked for the not-for-profit sector, but the number of comparisons in the for-profit sector is limited.

### Factors Contributing to a Quality Difference

Some of the qualitative studies (*n = *8) sought to explain the quality difference between the two sectors. Factors perceived to be related to low public-sector quality included resource constraints, low salaries, high workload, and poor incentives and conditions of service [39],[40],[44], the lack of a public family/general practice system that enables patients to return to the doctor(s) of their choice and develop relationships of trust over longer periods of time [43], public-sector drugs being sold privately [39],[40], staff favouring particular patients [39],[47], and clients lacking sufficient information about the appropriate use of drugs, resistance to antibiotics, costs, and their rights to challenge poor service [39],[46],[49],[54].

## Discussion

### Summary

The results of our analyses indicate that, in both private and public sectors, median values for structure, competence, and clinical practice fall around or below scores of 50/100. Whilst these values depend on the instruments used and the stringency of the primary research studies in applying these standards, the trends provide some insight into absolute performance, with obvious problems with technical aspects of care in both sectors.

In comparative performance, the formal private sector was better for drug availability, responsiveness, and effort. Overall, the median differences were modest, so stereotyped opinions that one sector is clearly better than another are not supported by this review.

Qualitative data portrayed formal private services that, in contrast to the public sector, were more client centred. This is consistent with the differences in care delivery shown by the quantitative data.

### Interpretation

In a formal private setting, drugs may be more available because funds are not restricted in the same way as in the public sector, and private providers are motivated to encourage patients to return, so responsiveness and effort are greater.

These results, combined with the fact that the private sector provides a substantial amount of health services, raise two further issues—the importance of paying attention to both sectors if overall quality is to be raised, and the need for governments to play a more active role in assuring quality of care.

Many efforts to improve the quality of ambulatory care are restricted to the public sector on the grounds that public funds should be reserved for the public sector because that is where the poor turn for their health care. But concentrating on the public sector misses a large proportion, the majority in some cases, of the providers used by the poor. Raising the quality of care delivered by private, as well as public, providers would, in fact, be a pro-poor intervention as it would improve the effectiveness of the money the poor spend on health care. A second argument advanced against spending public money on private providers is that because they provide a lower quality of care it is more effective to reserve funds for the public sector. The results of this review indicate that the overall quality of care from the two sets of providers is similar; if anything, the private sector is more responsive and drug availability is greater.

The overall low quality of care is likely to become even more so as the double burden of communicable disease (CD) and NCD becomes more prominent. Most health care providers, public or private, practicing today have been trained by institutions and work in health systems primarily oriented to CDs. Consequently, providers have only limited knowledge of NCDs, which demand a different set of clinical skills and a different approach to treatment. On most dimensions, effective treatment for NCDs requires approaches quite different to those that are available through the current health systems, and, contrary to views held by many, NCDs and associated risk factors are not the preserve of the rich; they are equally, if not more, prevalent among the poor [55]. Thus, it has to be considered that certain types of diseases, such as some NCDs, but also more complex CDs, such as AIDS, might require particularly high levels of structural quality, drug availability, and provider competence, while for other diseases, such as childhood diarrhoea, that are easy to diagnose and treat, it is most important to motivate providers to exert effort and practice what they already know [56].

Raising the quality of care in a health system is a long-term effort and requires attention to various aspects, including the incentive structure and training, both areas in which government has an important role, but to which it frequently pays little attention. Systematic and comprehensive traditional narrative reviews suggest a variety of strategies that can help increase quality. For example, supervision and audit with feedback, especially if combined with training, have been found to be effective [57]. However, an overall government bias against the private sector frequently means that too little attention is paid, and too few resources devoted, to overall supervision of the private sector. But setting standards, partly through ensuring standards of training, partly through licensing and accreditation of professionals (including emphasis on continuing education), and partly through consumer protection laws, is an important role of government [16],[58]. Researchers such as Leonard and colleagues [15] have provided useful theoretical frameworks for influencing the private sector based on the “principal-agent theory”. Others have proposed different ways of classifying the variety of strategies that have so far been used to improve the quality of private care, for example, classifying strategies according to the influence they have either on supply or demand or on the overall market environment [16],[59]. However, empirical evidence on the effectiveness of various approaches is somewhat limited, as the review by Peters et al. shows for reproductive health care [14].

### Strengths and Weaknesses of This Review

The search was comprehensive, the inclusion criteria were applied carefully, and quality criteria were applied to ensure comparisons were valid and were direct comparisons using the same methods. Given that studies used a very varied set of tools to measure quality of care, results on the absolute level of quality of care have to be interpreted with caution. However, results on the difference in quality of care can be interpreted with more confidence, because, as mentioned above, we took care to include only those studies that directly compared quality of care in the same country at the same time, using the same methods. A further strength is that we were able to categorise the various quality components to allow comparisons between studies. A disadvantage is that small studies could contribute as much to the estimates as large studies, but the sensitivity analysis—excluding the smaller studies—did not alter the direction of the differences between the sectors.

Although this review fully assessed eligible comparative studies on quality, additional work is needed to compare costs and aspects of equity. Similar to the dispute on quality, there are controversial views on whether private or public care is more costly or more accessible to the poor.

The review also highlights the lack of comparative evidence between the public sector and the private informal sector, although the latter is widely used [2],[60].

### Implications for Policy and Research

With the current evidence base, there is a clear need to consider quality of primary health services in both the public and private sector in order to improve health outcomes. There is a tendency for the private sector to provide better quality services, but further research on the overall quality and testing feasibility and effectiveness of mechanisms to improve quality will be critical for future health gains in LMICs.

Research needs to standardise outcomes and measures of socio-economic position across studies to improve comparability and to assist in between-country dialogue on effective quality assurance policies. Research on the effectiveness of market-led strategies to influence the private sector is important. Studies of dual practice, examining the same providers' behaviour in the two settings, could be useful specific studies in identifying factors in terms of the setting. Lastly, establishing minimum standards of care, and research to help identify effective approaches to achieve them, is central to achieving the health gains that are possible with current preventive and treatment medical technologies.

## Supporting Information

Alternative Language Abstract S1 Translation of the Abstract into French by Sima Berendes.
(0.02 MB DOCX)

Alternative Language Abstract S2 Translation of the Abstract into German by Sima Berendes.
(0.03 MB DOCX)

Table S1 Inclusion criteria for study reliability.
(0.07 MB DOC)

Table S2 Search strategy for Medline.
(0.07 MB DOC)

Table S3 Databases searched.
(0.05 MB DOC)

Table S4 Reasons for exclusion during screening of titles/abstracts or full papers.
(0.05 MB DOC)

Table S5 Excluded studies after reliability criteria application.
(0.09 MB DOC)

Table S6 Number of studies and comparisons per category.
(0.06 MB DOC)

Table S7 Results of comparisons between public and private providers for sub-Saharan Africa only and stratified by private provider type.
(0.06 MB DOC)

Table S8 Comparison of selected prescribing behaviours for patient visits to public-sector and formal for-profit private-sector care providers in LMICs.
(0.08 MB DOC)

Table S9 Characteristics of included quantitative studies summarised by world region.
(0.22 MB DOC)

## Abbreviations

CD: communicable disease
IQR: inter-quartile range
LMICs: low and middle income countries
NCD: non-communicable disease
NGO: non-governmental organization
WHO: World Health Organization
