# Supplementary material for: Quality of Private and Public Ambulatory Health Care in Low and Middle Income Countries: Systematic Review of Comparative Studies
Source: PLoS Med. 2011 Apr 12;8(4):e1000433. doi: 10.1371/journal.pmed.1000433 (PMC3075233; doi:10.1371/journal.pmed.1000433)
Supplement: Alternative Language Abstract S1 — Translation of the Abstract into French by Sima Berendes. (0.02 MB DOCX) [file pmed.1000433.s001.docx]

Translation of the abstract into French language by first author, Sima Berendes:

**La qualité des soins ambulatoires privés et publics dans les pays à revenu faible et intermédiaire: revue systématique des études comparatives**

**Résumé
Contexte**
Dans les pays en développement, le secteur privé offre une proportion importante des soins de santé primaires aux populations à faible revenu pour les maladies transmissibles et non-transmissibles. Les prestataires du secteur privé jouent donc un rôle crucial dans l’amélioration de l’état de santé de la population, et il est nécessaire de savoir comment leurs services se comparent avec ceux des prestataires du secteur public pour informer les options politiques.
**Méthodes et résultats**
Nous avons résumé des recherches fiables comparant la qualité des soins de santé ambulatoires formels privé ou public dans les pays à revenu faible et intermédiaire. Après une recherche exhaustive, nous avons choisi des études en fonction de critères d'inclusion, soit 80 études au total. Nous avons comparé la qualité en utilisant des catégories standardisées, converti les valeurs sur une échelle linéaire de 100%, calculé des différences entre les prestataires au sein de chaque étude, et résumé les valeurs médianes de ces différences à travers la totalité de ces études. Comme les résultats étaient semblables pour les services de soins à but lucratif et à but non-lucratif nous les avons combinés. Dans l'ensemble, les valeurs médianes ont indiqué que dans de nombreux services, qu’ils soient publics ou privés, les scores relatifs à l'infrastructure, les compétences cliniques et la pratique étaient bas. Dans l'ensemble, le secteur privé avait de meilleurs résultats en approvisionnement des médicaments, réactivité et effort. Aucune différence entre les groupes de prestataires n’a été détectée pour la satisfaction des patients ou la compétence. La synthèse des éléments qualitatifs indique que le secteur privé est plus orienté vers le client.
**Conclusions**
Bien que les données soient limitées, la qualité des soins ambulatoires parait pauvre dans les deux groupes de prestataires, le secteur privé donnant de meilleurs résultats sur la disponibilité des médicaments et certains aspects de prestation de soins, comme la réactivité et 'effort, et se tournant plus vers la clientèle. Les stratégies visant à influer sur la qualité des soins ambulatoires dans les deux groupes doivent améliorer la prestation et les résultats de soins offerts aux populations pauvres, y compris la gestion de la charge croissante des maladies non-transmissibles.
