# Supplementary material for: Quality of Private and Public Ambulatory Health Care in Low and Middle Income Countries: Systematic Review of Comparative Studies
Source: PLoS Med. 2011 Apr 12;8(4):e1000433. doi: 10.1371/journal.pmed.1000433 (PMC3075233; doi:10.1371/journal.pmed.1000433)
Supplement: Alternative Language Abstract S2 — Translation of the Abstract into German by Sima Berendes. (0.03 MB DOCX) [file pmed.1000433.s002.docx]

Translation of the abstract into German language by author, Sima Berendes.

**Qualität privater und öffentlicher ambulanter Gesundheitsversorgung in Ländern niedrigen und mittleren Einkommens: systematische Übersicht vergleichender Studien**

**Abstract**

**Hintergrund**
In Entwicklungsländern übernimmt der private Sektor einen erheblichen Teil der primären Gesundheitsversorgung für einkommensschwache Gruppen für übertragbare und nicht- übertragbare Krankheiten. Private Anbieter sind daher von zentraler Bedeutung für die Gesundheitsverbesserung der Bevölkerung. Es ist wichtig zu wissen, wie deren Dienstleistungen im Vergleich mit denen des öffentlichen Sektors abschneiden, um Information für politische Optionen zu generieren.
**Methoden und Ergebnisse**
Wir haben reliable Forschungsergebnisse zusammengefasst, die die Qualität formaler privater und öffentlicher ambulanter Gesundheitsversorgung in Ländern niedrigen und mittleren Einkommens vergleichen. Nach einer umfassenden Suche haben wir Studien nach Einschlusskriterien ausgewählt, was eine Auswahl von 80 Studien erbracht hat. Wir haben Qualität in Standard-Kategorien eingeteilt und verglichen, haben Ergebniswerte in eine lineare 100% Skala konvertiert, für jede Studie Unterschiede zwischen den Anbietern berechnet und Mediane der Unterschiede für alle Studien zusammengefasst. Da die Ergebnisse für Profit-und Non-Profit-Anbieter ähnlich waren, haben wir sie kombiniert. Insgesamt zeigten die Medianwerte, dass viele Dienstleistungen, unabhängig davon, ob sie öffentlich oder privat waren, niedrige Punktwerte hatten in Bezug auf Infrastruktur, klinische Kompetenz und Praxis. Insgesamt war der private Sektor überlegen in Bezug auf Medikamentenversorgung, Responsivität und Bemühen. Es konnte kein Unterschied zwischen den Anbietergruppen nachgewiesen werden bezüglich Patientenzufriedenheit oder Kompetenz. Die Synthese qualitativer Komponenten zeigte, dass der private Sektor Klienten-zentrierter ist.
**Schlussfolgerungen**
Trotz begrenzter Daten scheint die Qualität in beiden Gruppen schlecht zu sein, wobei der private Sektor bessere Leistung zu erbringen scheint hinsichtlich der Verfügbarkeit von Medikamenten und Aspekten der Art und Vermittlung von Gesundheitsversorgung, einschließlich Responsivität und Bemühen, und potentiell auch in der Kundenorientiertheit. Es sind Strategien erforderlich, die versuchen die Qualität beider Gruppen zu beeinflussen, um die Weise der Versorgung und Gesundheitsergebnisse für die die Armen zu verbessern, einschließlich des Zurechtkommens mit zunehmender Belastung durch nicht übertragbare Krankheiten.

Listen

Read phonetically

Dictionary - [View detailed dictionary](http://www.google.com/dictionary?source=translation&hl=en&q=Quality%20of%20private%20and%20public%20ambulatory%20health%20care%20in%20low%20and%20middle%20income%20countries:%20systematic%20review%20of%20comparative%20studies%20%20%20%20Abstract%20%20Background%20%20In%20developing%20countries,%20the%20private%20sector%20provides%20a%20substantial%20proportion%20of%20primary%20health%20care%20to%20low%20income%20groups%20for%20communicable%20and%20non-communicable%20diseases.%20These%20providers%20are%20therefore%20central%20to%20improving%20health%20outcomes.%20We%20need%20to%20know%20how%20their%20services%20compare%20to%20the%20public%20sector%20to%20inform%20policy%20options.%20%20%20Methods%20and%20findings%20%20We%20summarised%20reliable%20research%20comparing%20the%20quality%20of%20formal%20private%20versus%20public%20ambulatory%20health%20care%20in%20low%20and%20middle%20income%20countries.%20We%20selected%20studies%20against%20inclusion%20criteria%20following%20a%20comprehensive%20search,%20yielding%2080%20studies.%20We%20compared%20quality%20under%20standard%20categories;%20converted%20values%20to%20a%20linear%20100%25%20scale;%20calculated%20differences%20between%20providers%20within%20studies;%20and%20summarized%20median%20values%20of%20the%20differences%20across%20studies.%20As%20the%20results%20in%20for-profit%20and%20not-for-profit%20providers%20were%20similar%20we%20combined%20them.%20Overall,%20median%20values%20indicated%20that%20many%20services,%20irrespective%20of%20whether%20public%20or%20private,%20scored%20low%20on%20infrastructure,%20clinical%20competence%20and%20practice.%20Overall,%20the%20private%20sector%20performed%20better%20in%20relation%20to%20drug%20supply,%20responsiveness%20and%20effort.%20No%20difference%20between%20provider%20groups%20was%20detected%20for%20patient%20satisfaction%20or%20competence.%20Synthesis%20of%20qualitative%20components%20indicates%20the%20private%20sector%20is%20more%20client%20centred.%20%20%20%20Conclusions%20%20Although%20data%20are%20limited,%20quality%20in%20both%20provider%20groups%252)
