# Supplementary material for: Quality of Private and Public Ambulatory Health Care in Low and Middle Income Countries: Systematic Review of Comparative Studies
Source: PLoS Med. 2011 Apr 12;8(4):e1000433. doi: 10.1371/journal.pmed.1000433 (PMC3075233; doi:10.1371/journal.pmed.1000433)
Supplement: Table S1 — Inclusion criteria for study reliability. (0.07 MB DOC) [file pmed.1000433.s003.doc]

**Table S1. Inclusion criteria for study reliability**

| **Aspect** | **Category** | **Minimum criteria for inclusion** | **Classified as high quality** |
| --- | --- | --- | --- |
| Data collection in facilities | Sampling | Census of facilities or random selection from list. |  |
|  |  | Census of comparators, or comparators randomly selected, matched, or adjacent to index facilities. |  |
|  |  | Exclude if purposeful selection. |  |
|  | Sample size | At least five facilities per group | At least ten facilities per group |
| Data collection among patients | Sampling | Randomly or consecutively selected from a pre-defined starting point until reaching a pre-defined number or point in time. |  |
|  | Sample size | 20 patients or records per facility, or ≥ 100 per provider group. | ≥ 300 per provider group |
|  |  | For single conditions/ tracer conditions, or if narrowly defined age group, and/or if assessment through simulated patients ≥ 30 per condition or provider group |  |
| Data collection through community surveys | Sampling | Household surveys: Census of households or random selection from list. |  |
|  | Sample size | At least 1000 households should have been approached or total of ≥ 100 persons identified who sought care. | ≥ 600 persons identified who sought care |
|  |  | For studies using single conditions or tracer conditions, or focus on narrowly defined age group ≥ 30 patients per group. |  |
| Data collection on all levels | Response rate | Response rate required for questionnaires that were mailed to providers; indicated response rate should be at least 50%. | Indicated response rate should be at least 65%. |
| Data presentation | Description of basic data | Data and tables should add up and be consistent. | Relevant data should be very well described. |
|  |  | Depending on quality of other aspects, decide whether to include or exclude, if no absolute numbers given or if no denominators available for percentages. |  |
|  |  | Exclude if obvious data errors; inquire from authors in case of suspected typos. |  |
|  | Statistics | If statistical tests were performed, these need to be appropriate. |  |
| Other | Other aspects of design or analysis | No other important issues in design, conduct or analysis that could introduce bias considered on an individual basis, e.g. amount of potential bias if using different methods for collecting data between private and public providers |  |
|  | Issues related to conduct | No untoward events occurred during study that could introduce bias |  |
