# Supplementary material for: Quality of Private and Public Ambulatory Health Care in Low and Middle Income Countries: Systematic Review of Comparative Studies
Source: PLoS Med. 2011 Apr 12;8(4):e1000433. doi: 10.1371/journal.pmed.1000433 (PMC3075233; doi:10.1371/journal.pmed.1000433)
Supplement: Table S2 — Search strategy for Medline. (0.07 MB DOC) [file pmed.1000433.s004.doc]

**Table S2. Search strategy for Medline**

| 1 | Private Sector [Mesh] |
| --- | --- |
| 2 | Delivery of Health Care [Mesh] |
| 3 | Health sector [Mesh] |
| 4 | Primary health care [Mesh] |
| 5 | 1 and (2 or 3 or 4) |
| 6 | Private AND (health care OR healthcare OR practitioner* OR health provider* OR health provisio* OR medical care OR health clinic* OR outpatient service* OR ambulatory care OR hospital* OR pharmacy* OR drug vendor* OR drug seller* OR drug store) ti, ab |
| 7 | 5 or 6 |
| 8 | Public sector [Mesh] |
| 9 | Delivery of Health Care [Mesh] |
| 10 | Health sector [Mesh] |
| 11 | Primary health care [Mesh] |
| 12 | 8 AND (9 OR 10 OR 11) |
| 13 | Public AND (health care OR healthcare OR practitioner* OR health provider* OR health provisio*OR medical care OR health clinic* OR outpatient service* OR ambulatory care OR hospital* OR pharmacy* OR drug vendor* OR drug seller* OR drug store) ti, ab |
| 14 | 12 OR 13 |
| 15 | 7 OR 14 |
| 16 | Developing countries [Mesh] |
| 17 | Africa [Mesh] |
| 18 | Asia [Mesh] |
| 19 | South America [Mesh] |
| 20 | (less* developed countr*) OR (third world countr*) OR (under developed countr*) OR (underdeveloped countr*) OR (developing countr*) ti, ab |
| 21 | (low income countr*) OR (low income nation*) OR (middle income countr*) OR (middle income nation*) OR (low and middle income countr*) ti, ab |
| 22 | 16 OR 17 OR 18 OR 19 OR 20 OR 21 |
| 23 | 15 AND 22 |
| 24 | Quality of Health care [Mesh] |
| 25 | 23 AND 24 |
