# Supplementary material for: Quality of Private and Public Ambulatory Health Care in Low and Middle Income Countries: Systematic Review of Comparative Studies
Source: PLoS Med. 2011 Apr 12;8(4):e1000433. doi: 10.1371/journal.pmed.1000433 (PMC3075233; doi:10.1371/journal.pmed.1000433)
Supplement: Table S3 — Databases searched. (0.05 MB DOC) [file pmed.1000433.s005.doc]

***Table S3. Databases searched***

| - Medline (Pubmed) (1970- 6 April 2009) - Embase (1980 – 28 April 2009) - Psychinfo (1970 – 27 April 2009) - Lilacs (1982 – 27 April 2009) - Cochrane Central Register of Controlled Trials (CENTRAL), published in The Cochrane Library, issue 2 2009 - Cochrane Methods studies (issue 2 2009) - Cochrane economic evaluations (issue 2 2009) - Cochrane reviews (issue 2 2009) - Other reviews (in Cochrane library) - Web of Science, Social Sciences Citation Index and Science Citation index, (1900/14 to 2009) - CSA – Assia, Sociological Abstracts (1987 – 27 April 2009) - Econlit (1969 – 28 Oct 2010) |
| --- |
