# Supplementary material for: Quality of Private and Public Ambulatory Health Care in Low and Middle Income Countries: Systematic Review of Comparative Studies
Source: PLoS Med. 2011 Apr 12;8(4):e1000433. doi: 10.1371/journal.pmed.1000433 (PMC3075233; doi:10.1371/journal.pmed.1000433)
Supplement: Table S4 — Reasons for exclusion during screening of titles/abstracts or full papers. (0.05 MB DOC) [file pmed.1000433.s006.doc]

Table S4. Reasons for exclusion of studies during screening of titles/abstracts or full papers

| **Reason for exclusion during screening** | **No. of records** |
| --- | --- |
| Language other than English, French or German | 240 |
| Inappropriate design, i.e. not a study | 1049 |
| No LMIC or no within country comparison | 713 |
| No allopathic or no ambulatory health care | 4500 |
| No quality comparison between public and private providers | 1537 |
