# Supplementary material for: Quality of Private and Public Ambulatory Health Care in Low and Middle Income Countries: Systematic Review of Comparative Studies
Source: PLoS Med. 2011 Apr 12;8(4):e1000433. doi: 10.1371/journal.pmed.1000433 (PMC3075233; doi:10.1371/journal.pmed.1000433)
Supplement: Table S5 — Excluded studies after reliability criteria application. (0.09 MB DOC) [file pmed.1000433.s007.doc]

**Table S5. Excluded studies after reliability criteria application**

| **Citation** | **Reason for exclusion** |
| --- | --- |
| Abuaku, 2005 [1] | Low sample size of facilities (3 public and 2 private facilities), sampling of facilities unclear |
| Al-Azzam, 2007 [2] | Low sample size of comparable facilities (results separately reported for 3 major general public hospitals, 2 military hospitals, 3 private hospitals, 20 private clinics) |
| Ball, 2003 [3] | Response rate to self-administered questionnaires too low (42.4%) |
| Banerjee, 2003 [4] | Number of facilities and visits per provider group unclear. |
| Beattie, 1998 [5] | Risk of bias due to sampling: purposive sampling of facilities thought to offer above-average quality of care; since primary purpose of study was not to compare private vs. Public care, data presented in a way that made comparison difficult; facility sample size per ownership group unclear |
| Benjarattanaporn, 1997 [6] | Low sample size of public facilities and of private patients (6 private, but only 3 public clinics; 31 patients at private STD clinics, 29 at general private clinics, and 101 patients at public STD clinics) |
| Bhatia, 2004 [7] | Risk of bias through self-selection of private providers, because while 100% response rate of public providers, only < 50% response rate of private providers (census of 40 private providers invited to meeting, where 30 persons attended. Subsequently 20 were selected on basis of patient numbers and willingness to participate, of which 18 finally participated) |
| Bhatnagar, 2003 [8] | Low sample size with total of 30 prescriptions for public and private providers. |
| Doyle, 1998 [9] | Number of facilities and visits per provider group unclear. |
| Gilson, 1993 [10] | Sample size and number of facilities per provider group unclear. |
| Greenhalgh, 1987 [11] | Risk of bias due to sampling: Public hospitals and primary health care centres were selected following a kind of ‘snowball sampling’ (reported by author via email); for selection of private GPs the investigator “found a fairly poor residential area and walked around” until he found a GP’s surgery. |
| Huff-Rousselle, 2001 [12] | Low facility sample size (1 public and 1 private facility), no random sampling |
| Humayun, 2008 [13] | Low facility sample size (1 public and 1 private facility) |
| Hussein, 1997 [14] | Low facility sample size (3 public and 3 private hospitals) |
| Kloos, 1986 [15] | Low sample size of facilities (2 public and 2 private pharmacies); purposive sampling |
| Kumar, 2008 [16] | Risk of bias due to sampling: In rural areas stratified random sampling of blocks in districts, then census of public providers, but convenience sampling of private providers; in urban areas random sampling of public providers, for private providers two municipalities randomly chosen, then convenience sampling |
| Lundberg, 2007 | Sampling and number of interviews per provider group unclear |
| Maiga, 2003 [17] | Sampling and number of facilities per provider group unclear. |
| Mills, 1997 [18] | Low facility sample size (3 private and 3 public hospitals in South Africa, and 2 public and 2 private hospitals in Zimbabwe) |
| Mills, 2004 [19] | Risk of bias due to purposive sampling |
| Morris, 2007 [20] | Low patient sample size (total of 381 transport workers approached, out of whom 55 had sought treatment for STI symptoms and 45.1% = 25 attended private and 31.4%= 17 attended public facilities, remaining 23.5% sought treatment at a pharmacy ) |
| Ndyomugyenyi, 2007 [21] | Low sample size of public facilities (only 1 public facility) |
| Nshakira, 2002 [22] | Low sample size of facilities (2 private drug shops and 2 government district medical units); unclear sampling of facilities |
| Obaseiki-Ebor, 1987 [23] | Low sample size of facilities (5 private clinics, but only 2 government hospitals); unclear sampling |
| O’Hara, 2001 [24] | Low observation sample size for public providers (only total of 14 observations in 5 public facilities; the 121 observations in 14 “strengthened” public facilities cannot be considered, because these facilities had just received intervention) |
| Palmer, 2003 [25] | Low sample size of comparable facilities (5 public clinics, but only 2 private clinics run by company and 2 private GP practices); unclear sampling |
| Paphassarang, 2002 [26] | Low sample size of comparable facilities (20 private pharmacies, but only 4 public pharmacies), unclear sampling (private pharmacies randomly sampled, but not indicated how public pharmacies were selected) |
| Paredes, 1996 [27] | Risk of bias due to sampling: purposive sampling of providers within facilities; results not reported separately for different assessment methods |
| Phadke, 1996 [28] | Risk of bias due to purposive sampling |
| Pitaknetinan, 1999 [29] | Low sample size of comparable facilities (3 public, 3 private for-profit, and 3 private non-profit hospitals); purposive sampling |
| Schneider, 2002 [30] | Low sample size of facilities (5 private doctors, but only 2 public nurse-based health centres); sampling unclear |
| Sinanovic, 2006 [31] | Low sample size of comparable facilities (2 public and 2 private facilities); purposive sampling |
| Somse, 2000 [32] | Sampling of private providers unclear |
| Tangcharoensathien, 1999 [33] | Low sample size of comparable facilities (3 public, 3 private for-profit, and 3 private non-profit hospitals); purposive sampling |
| Turan, 2006 [34] | Low sample size of comparable facilities (1 MOH hospital, 1 social security hospital, and 1 private hospital); purposive sampling |
| Van Sta, 1996 [35] | Sampling unclear |
| Wilks, 2001 [36] | Low sample size of comparable facilities (2 public and 1 private clinic); no random sampling |

**References of Table S5**

1. Abuaku BK, Koram KA, Binka FN (2005) Antimalarial prescribing practices: a challenge to malaria control in Ghana. Medical principles and practice : international journal of the Kuwait University, Health Science Centre 14: 332-337.

2. Al-Azzam SI, Najjar RB, Khader YS (2007) Awareness of physicians in Jordan about the treatment of high blood pressure according to the seventh report of the Joint National Committee (JNC VII). European journal of cardiovascular nursing : journal of the Working Group on Cardiovascular Nursing of the European Society of Cardiology 6: 223-232.

3. Ball DE, Mazarurwi P (2003) HIV/AIDS knowledge and attitudes amongst pharmacists in Zimbabwe. The Central African journal of medicine 49: 27-31.

4. Banerjee A, Deaton A, Duflo E (2003) Health care delivery in rural Rajasthan. Poverty Action Lab Paper: Poverty Action Lab: Translating research into action.

5. Beattie A, Kalk WJ, Price M, Rispel L, Broomberg J, et al. (1998) The management of diabetes at primary level in South Africa: The results of a facility-based assessment. Journal of The Royal Society for the Promotion of Health118(6)()(pp 338-345), 1998Date of Publication: Dec 1998: 338-345.

6. Benjarattanaporn P, Lindan CP, Mills S, Barclay J, Bennett A, et al. (1997) Men with sexually transmitted diseases in Bangkok: where do they go for treatment and why? AIDS 11 Suppl 1: S87-95.

7. Bhatia J, Cleland J (2004) Health care of female outpatients in south-central India: comparing public and private sector provision. Health Policy and Planning 19: 402-409.

8. Bhatnagar T, Mishra CP, Mishra RN (2003) Drug prescription practices: a household study in rural Varanasi. Indian J Prev Soc Med 34: 33-39.

9. Doyle V, Castro H, Rojas Z, Sandiford P (1998) Public vs private sector in Central America: Consumer preception of quality. 3rd International Conference on Strategic Issues in Health Care Management. Scotland: St Andrews University.

10. Gilson L, Kitange H, Teuscher T (1993) Assessment of process quality in Tanzanian primary care. Health Policy26(2)()(pp 119-139), 1993Date of Publication: 1993: 119-139.

11. Greenhalgh T (1987) Drug prescription and self-medication in India: an exploratory survey. Soc Sci Med 25: 307-318.

12. Huff-Rousselle M, Pickering H (2001) Crossing the public-private sector divide with reproductive health in Cambodia: Out-patient services in a local NGO and the national MCH clinic. International Journal of Health Planning and Management16(1)()(pp 33-46), 2001Date of Publication: 2001: 33-46.

13. Humayun A, Fatima N, Naqqash S, Hussain S, Rasheed A, et al. (2008) Patients' perception and actual practice of informed consent, privacy and confidentiality in general medical outpatient departments of two tertiary care hospitals of Lahore. BMC medical ethics 9: 14.

14. Hussein AK, Mujinja PG (1997) Impact of user charges on government health facilities in Tanzania. East African medical journal 74: 751-757.

15. Kloos H, Chama T, Abemo D, Tsadik KG, Belay S (1986) Utilization of pharmacies and pharmaceutical drugs in Addis Ababa, Ethiopia. Soc Sci Med 22: 653-672.

16. Kumar R, Indira K, Rizvi A, Rizvi T, Jeyaseelan L (2008) Antibiotic prescribing practices in primary and secondary health care facilities in Uttar Pradesh, India. Journal of clinical pharmacy and therapeutics 33: 625-634.

17. Maiga FI, Haddad S, Fournier P, Gauvin L (2003) Public and private sector responses to essential drugs policies: a multilevel analysis of drug prescription and selling practices in Mali. Social science & medicine (1982) 57: 937-948.

18. Mills A, Hongoro C, Broomberg J (1997) Improving the efficiency of district hospitals: is contracting an option? Tropical medicine & international health : TM & IH 2: 116-126.

19. Mills A, Palmer N, Gilson L, McIntyre D, Schneider H, et al. (2004) The performance of different models of primary care provision in Southern Africa. Social science & medicine (1982) 59: 931-943.

20. Morris CN, Ferguson AG (2007) Sexual and treatment-seeking behaviour for sexually transmitted infection in long-distance transport workers of East Africa. Sexually transmitted infections 83: 242-245.

21. Ndyomugyenyi R, Magnussen P, Clarke S (2007) Malaria treatment-seeking behaviour and drug prescription practices in an area of low transmission in Uganda: implications for prevention and control. Transactions of the Royal Society of Tropical Medicine and Hygiene 101: 209-215.

22. Nshakira N, Kristensen M, Ssali F, Whyte SR (2002) Appropriate treatment of malaria? Use of antimalarial drugs for children's fevers in district medical units, drug shops and homes in eastern Uganda. Trop Med Int Health 7: 309-316.

23. Obaseiki-Ebor EE, Akerele JO, Ebea PO (1987) A survey of antibiotic outpatient prescribing and antibiotic self-medication. J Antimicrob Chemother 20: 759-763.

24. O'Hara HB, Voeten HA, Kuperus AG, Otido JM, Kusimba J, et al. (2001) Quality of health education during STD case management in Nairobi, Kenya. International journal of STD & AIDS 12: 315-323.

25. Palmer N, Mills A, Wadee H, Gilson L, Schneider H (2003) A new face for private providers in developing countries: what implications for public health? Bull World Health Organ 81: 292-297.

26. Paphassarang C, Philavong K, Boupha B, Bilas E (2002) Equity, privatisation and cost recovery in urban health care: the case of Lao PDR. . Health Policy and Planning 17: 72-84.

27. Paredes P, de la Pena M, Flores-Guerra E, Diaz J, Trostle J (1996) Factors influencing physicians' prescribing behaviour in the treatment of childhood diarrhoea: knowledge may not be the clue. Soc Sci Med 42: 1141-1153.

28. Phadke AR (1996) The quality of prescribing in an Indian district. Natl Med J India 9: 60-65.

29. Pitaknetinan K, Tangcharoensathien V, Supachutikul A, Bennett S, Mills A (1999) Profit, payment and pharmaceutical practices: perspectives from hospitals in Bangkok. Health policy (Amsterdam, Netherlands) 46: 179-194.

30. Schneider H, Palmer N (2002) Getting to the truth? Researching user views of primary health care. Health Policy Plan 17: 32-41.

31. Sinanovic E, Kumaranayake L (2006) Quality of tuberculosis care provided in different models of public-private partnership in South Africa. Int J Tuberc Lung Dis 10: 795-801.

32. Somse P, Mberyo-Yaah F, Morency P, Dubois MJ, Gresenguet G, et al. (2000) Quality of sexually transmitted disease treatments in the formal and informal sectors of Bangui, Central African Republic. Sexually transmitted diseases 27: 458-464.

33. Tangcharoensathien V, Bennett S, Khongswatt S, Supacutikul A, Mills A (1999) Patient satisfaction in Bangkok: the impact of hospital ownership and patient payment status. International journal for quality in health care : journal of the International Society for Quality in Health Care / ISQua 11: 309-317.

34. Turan JM, Bulut A, Nalbant H, Ortayli N, Akalin AA (2006) The quality of hospital-based antenatal care in Istanbul. Studies in family planning 37: 49-60.

35. Van Sta A, Hardon A (1996) Injection practices in the developing world: Results and recommendations from field studies in Uganda and Indonesia. WHO/DAP/964: World Health Organization.

36. Wilks RJ, Sargeant LA, Gulliford MC, Reid ME, Forrester TE (2001) Management of diabetes mellitus in three settings in Jamaica. Revista Panamericana de Salud Publica/Pan American Journal of Public Health9(2)()(pp 65-72), 2001Date of Publication: 2001: 65-72.
