# Supplementary material for: Quality of Private and Public Ambulatory Health Care in Low and Middle Income Countries: Systematic Review of Comparative Studies
Source: PLoS Med. 2011 Apr 12;8(4):e1000433. doi: 10.1371/journal.pmed.1000433 (PMC3075233; doi:10.1371/journal.pmed.1000433)
Supplement: Table S6 — Number of studies and comparisons per category. (0.06 MB DOC) [file pmed.1000433.s008.doc]

Table S6. Number of studies and comparisons per category

| Category | Component | Studies | Comparisons | Comparisons converted to linear scale |
| --- | --- | --- | --- | --- |
|  |  | (No.) | (No.) | (No.) |
| Structural | Building, equipment & material | 22 | 27 | 26 |
|  | Drug availability | 14 | 14 | 14 |
| Delivery | Responsiveness | 10 | 13 | 7 |
|  | Effort | 7 | 7 | 3 |
|  | Patient satisfaction | 8 | 10 | 10 |
| Technical | Competence | 18 | 21 | 19 |
|  | Clinical practice | 34 | 41 | 22 |
