# Supplementary material for: Quality of Private and Public Ambulatory Health Care in Low and Middle Income Countries: Systematic Review of Comparative Studies
Source: PLoS Med. 2011 Apr 12;8(4):e1000433. doi: 10.1371/journal.pmed.1000433 (PMC3075233; doi:10.1371/journal.pmed.1000433)
Supplement: Table S7 — Results of comparisons between public and private providers for sub-Saharan Africa only and stratified by private provider type. (0.06 MB DOC) [file pmed.1000433.s009.doc]

**Table S7. Results of comparisons between public and formal private providers for Sub-Saharan Africa only and stratified by private provider type**

| **Category** | **Sub-Category** | **Private For-Profit (FP) vs Public** | | | **Private Not-for-Profit (NP) vs Public** | | | **Private FP & NP vs Public** | | |
| --- | --- | --- | --- | --- | --- | --- | --- | --- | --- | --- |
|  |  | **Comp. conv. to 100% scale (n)** | **Median diff.*** | **IQR**  **diff.** | **Comp. conv. to 100% scale (n)** | **Median diff.*** | **IQR**  **diff.** | **Comp. conv. to 100% scale (n)** | **Median diff.*** | **IQR**  **diff.** |
| Structural | Building, equipment, material | 5 | -0.5 | -7.0,4.2 | 10 | 6.0 | -7.0,4.2 | 2 | 2.5 | -3.3,8.3 |
|  | Drug availability | 5 | 15.0 | 12.3, 20.7 | 1 | 35.0 | 35.0, 35.0 | 0 | n/a | n/a |
| Delivery | Responsiveness | 1 | 7.0 | 7.0 | 3 | 9.0 | 6.3,15.5 | 1 | 7.0 | 7.0, 7.0 |
|  | Effort | 1 | 8.0 | 8.0, 8.0 | 0 | n/a | n/a | 1 | 3.0 | 3.0, 3.0 |
|  | Patient satisfaction | 2 | 1.5 | 0.8, 2.3 | 4 | -1.4 | -4.5, 6.0 | 0 | n/a | n/a |
| Technical | Competence | 6 | -6.2 | -14.0, -1.1 | 3 | -3.0 | -21.5, -1.0 | 1 | -17.5 | -17.5, -17.5 |
|  | Clinical practice | 4 | 21.3 | 12.3, 27.9 | 8 | 5.8 | 2.4,12.1 | 3 | 11.5 | 8.2,13.0 |
| Across all (sub-)categories | | 24 | 4.1 | -5.1,15.0 | 29 | 3.6 | -0.5,12.5 | 8 | 5.9 | 0.0,12.1 |

* positive value means private sector better
