# Supplementary material for: Quality of Private and Public Ambulatory Health Care in Low and Middle Income Countries: Systematic Review of Comparative Studies
Source: PLoS Med. 2011 Apr 12;8(4):e1000433. doi: 10.1371/journal.pmed.1000433 (PMC3075233; doi:10.1371/journal.pmed.1000433)
Supplement: Table S8 — Comparison of selected prescribing behaviours for patient visits to public-sector and formal for-profit private-sector care providers in LMICs. (0.08 MB DOC) [file pmed.1000433.s010.doc]

**Table S8 Comparison of selected prescribing behaviour for patient visits in public and formal private for-profit sector in LMIC**

| **Citation** | **Country** | **Type of condition** | **Mean no. of drugs** | | **% Antibiotics** | | **% Injections** | | **% ORS** | |
| --- | --- | --- | --- | --- | --- | --- | --- | --- | --- | --- |
|  |  |  | **Publ.** | **Priv.** | **Publ.** | **Priv.** | **Publ.** | **Priv.** | **Publ.** | **Priv.** |
| Abdo-Rabbo, 2003 [1] | Yemen | Malaria | 3 | 4 |  |  | 17.2 | 33.5 |  |  |
| Ahmed, 1996 [2] | Tanzania | Various | ≥ 3 in 59% | ≥ 3 in 81% |  |  |  |  |  |  |
| Bhatia, 2001 [3] | India | Various |  |  |  |  | 82.8 | 84.7 |  |  |
| Commeyras, 2006 [4] | Cameroon | Various | 4.6 | 4.5 |  |  |  |  |  |  |
| Langsten, 1995 [5] | Egypt | Childhood diarrhoea |  |  | 44.2 | 65.4 |  |  | 72.6 | 58.8 |
| Lindelow, 2003 [6] | Uganda | Various |  |  | 48 | 41 |  |  |  |  |
| Maiga, 2006 [7] | Mali | Various | 3.2 | 2.8 | 70.4 | 50 | 33.2 | 14.3 |  |  |
| Massele, 1997 [8] | Tanzania | Various | 2.2 | 2.5 | 12.3 | 19.7 | 9.6 | 12.7 |  |  |
| Muhuri, 1996 [9] | Six countries | Childhood diarrhoea |  |  |  |  |  |  | 61.2 | 49.6 |
| Ogwal-Okeng, 2004 [10] | Uganda | ARI | 2.9 | 3.1 | 91.3 | 88 | 14.3 | 7.7 |  |  |
|  |  | Malaria | 3.3 | 3.1 |  |  |  |  |  |  |
| Patel, 2005 [11] | India | Various | 2.4 | 2.9 | 14.5 | 28.2 |  |  |  |  |
| Pongsupap, 2006 [12] | Thailand | Stomach ache | 2.8 | 3.8 |  |  |  |  |  |  |
| Siddiqi, 2002 [13] | Pakistan | Various | 2.7 | 4.1 | 54 | 63 | 22 | 48 |  |  |
| Simon, 1998 [14] | Morocco | Various | 2.4 | 3.4 | about 44 | about 44 | about 17 | about 17 |  |  |
| Waters, 2008 [15] | Six countries | Childhood diarrhoea |  |  |  |  |  |  | 33 | 13.7 |

Note: ORS = oral rehydration salts

**References of table S8**

1. Abdo-Rabbo A (2003) Prescribing rationality and availability of antimalarial drugs in Hajjah, Yemen. Eastern Mediterranean health journal = La revue de sante de la Mediterranee orientale = al-Majallah al-sihhiyah li-sharq al-mutawassit 9: 607-617.

2. Ahmed AM, Urassa DP, Gherardi E, Game NY (1996) Patients' perception of public, voluntary and private dispensaries in rural areas of Tanzania. East African medical journal 73: 370-374.

3. Bhatia JC, Cleland J (2001) Health-care seeking and expenditure by young Indian mothers in the public and private sectors. Health policy and planning 16: 55-61.

4. Commeyras C, Ndo JR, Merabet O, Kone H, Rakotondrabe FP (2006) [Health and drug consumption profile in Cameroon]. Sante (Montrouge, France) 16: 13-19.

5. Langsten R, Hill K (1995) Treatment of childhood diarrhea in rural Egypt. Soc Sci Med 40: 989-1001.

6. Lindelow M, Reinikka R, Svensson J (2003) Health care on the front lines: Survey evidence on public and private providers in Uganda. 38. Washington DC: Human Development Sector, Africa Region, World Bank.

7. Maiga D, Diawara A, Maiga MD (2006) [Evaluation of rational prescribing and dispensing of medicines in Mali]. Rev Epidemiol Sante Publique 54: 497-505.

8. Massele AY, Nsimba SE (1997) Comparison of drug utilisation in public and private primary health care clinics in Tanzania. East African medical journal 74: 420-422.

9. Muhuri PK, Anker M, Bryce J (1996) Treatment patterns for childhood diarrhoea: evidence from demographic and health surveys. Bulletin of the World Health Organization 74: 135-146.

10. Ogwal-Okeng JW, Obua C, Waako P, Aupont O, Ross-Degnan D (2004) A comparison of prescribing practices between public and private sector physicians in Uganda. East African medical journal Suppl: S12-16.

11. Patel V, Vaidya R, Naik D, Borker P (2005) Irrational drug use in India: a prescription survey from Goa. Journal of postgraduate medicine 51: 9-12.

12. Pongsupap Y, Van Lerberghe W (2006) Choosing between public and private or between hospital and primary care: responsiveness, patient-centredness and prescribing patterns in outpatient consultations in Bangkok. Tropical medicine & international health : TM & IH 11: 81-89.

13. Siddiqi S, Hamid S, Rafique G, Chaudhry SA, Ali N, et al. (2002) Prescription practices of public and private health care providers in Attock District of Pakistan. The International journal of health planning and management 17: 23-40.

14. Simon N, Hakkou F, Minani M, Jasson M, Diquet B (1998) [Drug prescription and utilization in Morocco]. Therapie 53: 113-120.

15. Waters HR, Hatt LE, Black RE (2008) The role of private providers in treating child diarrhoea in Latin America. Health Economics 17: 21-29.
