# Supplementary material for: Quality of Private and Public Ambulatory Health Care in Low and Middle Income Countries: Systematic Review of Comparative Studies
Source: PLoS Med. 2011 Apr 12;8(4):e1000433. doi: 10.1371/journal.pmed.1000433 (PMC3075233; doi:10.1371/journal.pmed.1000433)
Supplement: Table S9 — Characteristics of included quantitative studies summarised by world region. (0.22 MB DOC) [file pmed.1000433.s011.doc]

Table S9. Characteristics of included quantitative studies summarized by world region

| ***East Asia and Pacific*** | | | |  |  |  |
| --- | --- | --- | --- | --- | --- | --- |
| **Study** | | **Country** | **Comparison** | **Quality Category** | **Sub-Category** | **Method** |
| Babar, 2007 [1] | | Malaysia | 20 public general hospitals vs. 32 private retail pharmacies | Structural | Drug availability | Observations * |
| Barber, 2007 [2] | | Indonesia | 582 public health centres (staffed on average by 1.43 doctors) vs. 523 private MDs | Structural | Building, equipment, material | Observations * |
|  | |  | 577 public health centres (staffed on average by 1.43 doctors) vs. 241 private MDs | Technical | Competence | Case scenarios/ vignettes * |
| Garner, 1990 [3] | | Papua New Guinea | 36 public clinics (26 health centres & 10 sub-centres) vs. 35 church clinics (12 health centres & 23 sub-centres) | Structural | Building, equipment, material | Observations * |
| Lim, 2004 [4] | | China | 1114 visitors of public vs. 767 visitors of private clinics | Delivery | Patient satisfaction | Household survey * |
| Liu, 2006 [5] | | China | 1555 visitors of non-private vs. 147 visitors of private providers | Delivery | Patient satisfaction | Household survey * |
| Meng, 2000 [6] | | China | 24 public vs. 23 private village clinics | Structural | Building, equipment, material | Observations * |
|  | |  |  | Technical | Competence | Case scenarios/ vignettes |
| Peters, 1991 [7] | | Philippines | 27 public and 11 private clinics for vaccine provision and 12 public and 10 private clinics for vaccine storage | Structural | Building, equipment, material | Observations * |
|  | |  | 27 public and 21 private clinics | Technical | Competence | Case scenarios/ vignettes * |
| Pongsupap, 2006 [8] | | Thailand | 91 public clinics and hospitals vs. 120 private clinics and hospitals | Delivery | Effort | Stand. Patient |
|  | |  |  | Delivery | Responsiveness | Stand. Patient * |
|  | |  |  | Technical | Clinical practice | Stand. Patient |
| Prempree, 2007 [9] | | Thailand | 18 government vs. 8 private clinics/hospital OPDs (36 STI patients were reported in private clinics and 52 in public health centres) | Technical | Clinical practice | Medical Record or Prescription * |
| Sihavong, 2007 [10] | | Lao PDR | 52 public providers (in 2 district hospitals and 11 health centres) and 36 private providers (in 22 clinics) | Technical | Competence | Case scenarios/ vignettes * |
| Syhakhang, 2001 [11] | | Lao PDR | 13 public vs. 92 private pharmacies | Structural | Drug availability | Observations * |
| (Tuan, 2005 [12] | | Vietnam | 30 public commune health centres vs. 182 private providers (priv. incl. 8% traditional practitioners, 11% no professional qualification, 12% pharmacists, remaining providers were doctors (9%) or other health staff – 53 of priv. providers were publ.-priv. mix) | Structural | Building, equipment, material | Observations |
|  | |  |  | Technical | Competence | Case scenarios/ vignettes |
|  | |  | 43 patients who had visited a public commune health centre vs. 110 patients who visited any type of private provider  *Note: since it was one of the two studies including informal private providers results were not included in analysis* | Delivery | Patient satisfaction | Household survey ) |
| ***Europe and Central Asia*** | | |  |  |  |  |
| **Study** | **Country** | | **Comparison** | **Quality Category** | **Sub-Category** | **Method** |
| Nordyke, 2002 [13] | Macedonia, FYR | | 73 public vs. 37 private primary care physicians | Structural | Building, equipment, material | Observations * |
|  |  | |  | Technical | Competence | Case scenarios/ vignettes * |
| ***Latin America and the Caribbean*** | | | |  |  |  |
| **Study** | **Country** | | **Comparison** |  | **Category** | **Method** |
| Barber, 2006 [14] | Mexico | | 1253 women identified during survey of 16 125 households: 737 women who visited government facilities vs. 234 women who visited private clinics | Technical | Clinical practice | Patient interview * |
| Barber, 2007 [15] | Mexico | | MDs in MOH and other government-operated health facilities (1,828 patient visits) vs. MDs working in the private sector (777 patient visits) | Technical | Clinical practice | Household survey * |
| Bojalil, 1998 [16] | Mexico | | 40 public and 41-59 (depending on condition) private GPs | Technical | Clinical practice | Observations * |
| Lewis, 2004 [17] | El Salvador | | Public vs. Private health facilities providing primary health care (total of 38 facilities) | Delivery | Responsiveness | Patient interview |
| Peabody, 1994 [18] | Jamaica | | 338 public (262 basic and 76 higher-level) and 159 private primary care clinics | Structural | Building, equipment, material | Observation * |
|  |  | |  | Technical | Clinical practice | Prov. Interview * |
| Waters, 2008 [19] | Ecuador, Guatemala, Nicaragua, Panama, Peru, Bolivia | | 2643 public clinics and hospitals vs. 1541 private hospitals, clinics, and pharmacies | Technical | Clinical practice | Household survey |
| ***Middle East and North Africa*** | | | |  |  |  |
| **Study** | **Country** | | **Comparison** | **Quality Category** | **Sub-Category** | **Method** |
| Abdo-Rabbo, 2003 [20] | Yemen, Rep. | | 17 public vs. 6 private facilities | Technical | Clinical practice | Medical Record or Prescription |
| Aounallah Skhiri, 2005 [21] | Tunisia | | 241 public GPs and 134 private GPs | Technical | Competence | Prov. Interview * |
| Langsten, 1995 [22] | Egypt, Arab Rep. | | 609 reported visits to government health facilities vs. 1873 visits to private physicians | Technical | Clinical practice | Household survey |
| MOH, 2003 [23] | Egypt, Arab Rep. | | 544 public facilities vs. 106 NGO facilities | Structural | Building, equipment, material | Observations * |
|  |  | | 3123 (incl. child care, ANC and STI) consultations in 544 public facilities vs. 311 (incl. child care, ANC and STI) consultations in 106 NGO facilities | Technical | Clinical practice | Observations * |
| MOH, 2005 [24] | Egypt, Arab Rep. | | 559 public vs. 100 NGO health facilities | Structural | Building, equipment, material | Observations * |
|  |  | | 3460 (incl. child care, ANC and STI) consultations in 559 public facilities vs. 326 (incl. child care, ANC and STI) consultations in 100 NGO facilities | Technical | Clinical practice | Observations * |
| Nikniyaz, 2006 [25] | Iran, Islamic Rep. | | Total of 1000 households visiting 9 private cooperative health centres vs. 18 public health centres | Delivery | Patient satisfaction | Household survey * |
| Simon, 1998 [26] | Morocco | | 120 prescriptions from public vs. 480 prescriptions from private providers | Technical | Clinical practice | Medical Record or Prescription |
| ***South Asia*** |  | |  |  |  |  |
| **Study** | **Country** | | **Comparison** | **Quality Category** | **Sub-Category** | **Method** |
| Akin, 1999 [27] | Sri Lanka | | 44 public facilities vs. 57 private facilities | Structural | Drug availability | Observations * |
| Bhatia, 2001 [28] | India | | 466 consultations with private and 123 consultations with public practitioners | Technical | Clinical practice | Household survey |
|  |  | |  | Delivery | Effort | Household survey * |
| Das, 2007 [29] | India | | 43 Public MBBS vs. 68 private MBBS | Technical | Competence | Case scenarios/ vignettes |
| IIPS, 2000 [30] | India | | 18,306 women who visited public health facility during last 12 months vs. 33,511 who visited private sector/NGO/Trust. | Structural | Building, equipment, material | Household survey * |
|  |  | |  | Delivery | Responsiveness | Household survey * |
| Janjua, 2007 [31] | Pakistan | | 29 public physicians vs. 126 private GPs | Technical | Competence | Prov. Interview * |
| Kotwani, 2003 [32] | India | | 20 public health facilities vs. 20 private retail pharmacies | Structural | Drug availability | Observations * |
| Kotwani, 2009 [33] | India | | Total of 134 public facilities vs. 170 private facilities/ retail pharmacies in four states | Structural | Drug availability | Observations * |
| Kumar, 2005 [34] | India | | 41 government dispensary doctors vs. 43 private practitioners | Technical | Competence | Formal test * |
| Mahaprata, 2003 [35] | India | | 554 exit interviews in 71 private clinics vs. 521 exit interviews in 53 public health centre | Delivery | Patient satisfaction | Patient interview * |
| Patel, 2005 [36] | India | | Public physicians (who issued 159 prescriptions) vs. Private physicians (who issued 831 prescriptions) | Technical | Clinical practice | Medical Record or Prescription |
| Siddiqi, 2002 [37] | Pakistan | | 60 public and 48 private facilities | Structural | Drug availability | Observations * |
|  |  | | 60 public and 48 private facilities (925 private vs. 988 public prescriptions) | Technical | Clinical practice | Observations |
|  |  | |  | Delivery | Effort | Observations |
| Vandan, 2008 [38] | India | | 79 public and 62 private physicians | Technical | Competence | Prov. Interview * |
| ***Sub-Saharan Africa*** | | |  |  |  |  |
| **Study** | **Country** | | **Comparison** | **Quality Category** | **Sub-Category** | **Method** |
| Ahmed, 1996 [39] | Tanzania | | 109 patients attending public vs. 107 patients attending private for-profit dispensaries | Delivery | Responsiveness | Patient interview |
|  |  | |  | Delivery | Patient satisfaction | Patient interview * |
|  |  | |  | Technical | Clinical practice | Patient interview |
|  |  | | 109 patients attending public vs. 104 patients attending NGO dispensaries | Delivery | Responsiveness | Patient interview |
|  |  | |  | Delivery | Patient satisfaction | Patient interview * |
|  |  | |  | Technical | Clinical practice | Patient interview |
| Akin, 1995 [40] | Nigeria | | Public versus private health facilities (total of n=86) | Structural | Drug availability | Observations * |
| Akoria, 2008 [41] | Nigeria | | 8 public vs. 9 private hospitals (746 vs. 451 prescriptions) | Technical | Clinical practice | Medical Record or Prescription |
| Bitran, 1995 [42] | Senegal | | 23 government health centres vs. 6 for-profit dispensaries | Structural | Building, equipment, material | Observations * |
|  |  | |  | Technical | Clinical practice | Observations * |
|  |  | |  | Delivery | Patient satisfaction | Patient interview * |
|  |  | | 46 government health posts vs. 30 catholic health posts | Structural | Building, equipment, material | Observations * |
|  |  | |  | Technical | Clinical practice | Observations * |
|  |  | |  | Delivery | Patient satisfaction | Patient interview * |
| Boller, 2003 [43] | Tanzania | | 7 public and 9 private first-tier providers offering mother and child health care | Structural | Building, equipment, material | Observations * |
|  |  | |  | Delivery | Responsiveness | Observations * |
|  |  | |  | Delivery | Effort | Observations * |
|  |  | |  | Technical | Clinical practice | Observations * |
| Cheraghali, 2009 [44] | Sudan | | 36 public health centres vs. 36 private pharmacies | Structural | Drug availability | Observations * |
| Commeyras, 2006 [45] | Cameroon | | 202 drug prescriptions by public health centres (‘centre medical d’ arrondissement’) vs. 402 prescriptions by private for-profit providers | Technical | Clinical practice | Patient interview |
|  |  | | 202 drug prescriptions by public health centres (‘centre medical d’ arrondissement’) vs. 268 prescriptions by private non-profit providers | Technical | Clinical practice | Patient interview |
| Fawole, 2008 [46] | Nigeria | | 57 health workers in 5 public and 230 health workers in 37 private primary health care facilities | Technical | Competence | Prov. Interview * |
| Gilson, 1995 [47] | Tanzania | | 40 government vs. 14 church dispensaries | Structural | Building, equipment, material | Observations * |
| GSS, 2003 [48] | Ghana | | 288 public vs. 39 private religious health facilities | Structural | Building, equipment, material | Observations * |
|  |  | | 2294 (child care and ANC) consultations in 288 public facilities vs. 602 (child care and ANC) consultations in 39 private religious facilities | Technical | Clinical practice | Observations * |
| Huebner, 1997 [49] | Botswana | | 95 government hospital physicians vs. 27 private hospital physicians | Technical | Clinical practice | Prov. Interview * |
| Kanji, 1995 [50] | Tanzania | | 28 government facilities vs. 15 voluntary facilities (catholic, protestant and muslim groups) | Structural | Building, equipment, material | Observations * |
|  |  | |  | Delivery | Responsiveness | Observations * |
|  |  | |  | Delivery | Effort | Observation |
|  |  | |  | Delivery | Patient satisfaction | Patient interview * |
|  |  | |  | Technical | Clinical practice | Observations * |
| Leonard, 2007 [51] | Tanzania | | Public doctors (in 22 facilities) vs. NGO doctors (in 12 facilities) | Technical | Competence | Case scenarios/ vignettes * |
|  |  | |  | Technical | Clinical practice | Observations * |
|  |  | | Public doctors (in 22 facilities) vs. Private for-profit doctors (in 5 facilities) | Technical | Competence | Case scenarios/ vignettes * |
|  |  | |  | Technical | Clinical practice | Observations * |
| Lindelow, 2003 [52] | Uganda | | Patients who visited 81 government vs. 30 private for-profit dispensaries (about 10 patients per facility) | Delivery | Responsiveness | Patient interview * |
|  |  | |  | Technical | Clinical practice | Patient interview |
|  |  | | Patients who visited 81 government vs. 44 non-profit (religious and NGO) dispensaries (about 10 patients per facility) | Delivery | Responsiveness | Patient interview * |
|  |  | |  | Technical | Clinical practice | Patient interview |
| Maiga, 2006 [53] | Mali | | 30 public primary health centres vs. 30 private pharmacies | Technical | Clinical practice | Medical Record or Prescription |
| Mariko, 2003 [54] | Mali | | Public hospitals and dispensaries versus private non-profit facilities (total stratified random sample of 42 out of 84 facilities visited by 1104 persons interviewed in 1191 household surveys, this comparison includes 3 out of 4 facility types, since it omits for-profit providers; 150 patients per facility) | Delivery | Responsiveness | Observation |
|  |  | |  | Technical | Clinical practice | Observation * |
|  |  | | Public hospitals and dispensaries versus private for-profit facilities (total stratified random sample of 42 out of 84 facilities visited by 1104 persons interviewed in 1191 household surveys, this comparison includes 3 out of 4 facility types, since it omits non-profit providers; 150 patients per facility) | Delivery | Responsiveness | Observation |
|  |  | |  | Technical | Clinical practice | Observation * |
| (Massele, 1993 [55] | Tanzania | | 20 public dispensaries vs. 20 informal private drug sellers | Structural | Drug availability | Observations |
|  |  | | 20 rural medical aids in public dispensaries vs. 20 informal private drug sellers  *Note: since this was one of the two studies including informal private providers results were not included in analysis.* | Technical | Competence | Prov. Interview) |
| Massele, 1997 [56] | Tanzania | | 20 public vs. 20 private outpatient clinics | Technical | Clinical practice | Medical Record or Prescription |
| Mbanefoh, 2004 [57] | Nigeria | | 1019 consultations in public vs 284 in private clinics | Delivery | Responsiveness | Household survey |
|  |  | | 1006 consultations in public vs 272 in private clinics | Delivery | Effort | Household survey |
| Mliga, 2003 [58] | Tanzania | | 16 government hospitals and health centres/ dispensaries vs. 15 Lutheran, 15 Catholic, and 7^th^ Day Adventists hospitals and health centres/ dispensaries | Structural | Drug availability | Patient interview * |
|  |  | |  | Delivery | Responsiveness | Observation * |
|  |  | |  | Delivery | Patient satisfaction | Patient interview * |
|  |  | |  | Technical | Clinical practice | Observation * |
| MOH, 2000 [59] | Kenya | | 364 sick children in 221 public vs. 94 sick children in 70 mission facilities | Technical | Clinical practice | Observations * |
|  |  | | 364 sick children in 221 public vs. 166 sick children in 95 other private facilities | Technical | Clinical practice | Observations * |
| MOH, 2003 [60] | Rwanda | | Child health facilities: 135 public vs. 71 Government-assisted (GA) NGOs; ANC facilities: 129 public vs. 70 GA-NGOs; STI facilities: 141 public vs. 79 GA-NGOs; | Structural | Building, equipment, material | Observations * |
|  |  | | Child health facilities: 1272 children in 135 public vs. 859 children in 71 Government-assisted (GA) NGOs; ANC facilities: 978 clients in 129 public vs. 556 clients in 70 GA-NGOs | Technical | Clinical practice | Observations * |
| MOHSW, 2007 [61] | Tanzania | | 477 public vs. 283 NGO clinics | Structural | Building, equipment, material | Prov. Interview * |
|  |  | | 477 public vs 162 private for-profit clinics | Structural | Building, equipment, material | Prov. Interview * |
| Mouyokani, 1999 [62] | Congo, Rep | | 1398 patients who visited 16 public health centres vs. 86 patients who visited 5 private nurses’ centres | Delivery | Effort | Patient interview * |
| Mwabu, 1993 [63] | Kenya | | 8 government health centres or dispensaries vs. 5 private clinics | Structural | Drug availability | Observations * |
| Nahum, 2000 [64] | Benin | | 71 private and 23 public medical doctors | Technical | Competence | Prov. Interview * |
| NBS, 2007 [65] | Tanzania | | ANC facilities: 385 government vs. 30 private for-profit; STI facilities: 385 government vs. 102 private for-profit | Structural | Building, equipment, material | Observations * |
|  |  | | ANC facilities: 385 government vs. 77 faith-based; STI facilities: 385 government vs. 87 faith-based | Structural | Building, equipment, material | Observations * |
| NCAPD, 2005 [66] | Kenya | | Total of 246 public vs. 63 private for-profit health facilities | Structural | Building, equipment, material | Observations * |
|  |  | | Total of 246 public vs. 131 private non-profit (21 NGOs, 110 FBOs) health facilities | Structural | Building, equipment, material | Observations * |
| Nhachi, 1993 [67] | Zimbabwe | | 47 public hospitals/ clinics (pharmacists/technicians/nurses/district medical officers) vs. 7 private hospitals/ clinics | Structural | Drug availability | Prov. Interview * |
| NIS, 2008 [68] | Rwanda | | 274 government facilities providing ANC and 304 government facilities providing STI services vs 120 government-assisted (GA) private religious and non-profit facilities providing ANC and 304 government-assisted (GA) private religious and non-profit facilities offering STI services | Structural | Building, equipment, material | Observations * |
|  |  | | 274 government facilities providing ANC and 304 government facilities providing STI services vs. 38 private/NGO/Community facilities providing ANC and 80 private/NGO/Community facilities offering STI services | Structural | Building, equipment, material | Observations * |
| Nshuti, 2001 [69] | Uganda | | 10 public versus 104 private clinics | Technical | Competence | Prov. Interview * |
| Ogwal-Okeng, 2004 [70] | Uganda | | 117 private physicians vs. 10 public health units | Technical | Clinical practice | Medical records or prescription and simulated patients |
| Pappas, 2004 [71] | Uganda | | 73 government vs. 21 non-government parish clinics (Health Center Grade II for outpatient care) | Structural | Building, equipment, material | Observation and Prov. Interview * |
| Richard, 2004 [72] | Chad | | 15 public and 9 private missionary providers | Structural | Building, equipment, material | Observations |
| Robertson, 2009 [73] | 14 countries in Sub-Saharan Africa | | 3 public primary health care clinics vs. 5 private or retail pharmacies in each country | Structural | Drug availability | Observations * |
| Sondo, 1999 [74] | Burkina Faso | | 85 public and 68 private providers from total of 112 health facilities (mostly primary, but also secondary, 1 tertiary) | Technical | Competence | Prov. Interview * |
| Suleiman, 2003 [75] | Somalia | | 7 exclusively public vs. 17 exclusively private medical doctors | Technical | Competence | Prov. Interview * |
| Tann, 2007 [76] | Uganda | | 41 women who had visited public vs. 45 women who had visited private hospitals for antenatal care | Technical | Clinical practice | Household survey * |
| Voeten, 2001 [77] | Kenya | | 9 public non-strengthened providers vs. 80 private providers in private for-profit clinics | Technical | Competence | Case scenarios/ vignettes * |
|  |  | | 9 public non-strengthened providers vs. 19 private providers in NGO/ community based clinics | Technical | Competence | Case scenarios/ vignettes * |
|  |  | | 9 public non-strengthened providers vs. 17 private providers in mission clinics | Technical | Competence | Case scenarios/ vignettes * |
| Vuylsteke, 2004 [78] | Côte d’Ivoire | | 13 public vs. 13 private health care facilities offering STI services | Structural | Building, equipment, material | Observations * |
| ***More than one region*** | | |  |  |  |  |
| **Study** | **Country** | | **Comparison** | **Quality Category** | **Sub-Category** | **Method** |
| Cameron, 2009 [79] | 36 countries in LMIC | | 36 surveys for public sector, 40 surveys for private sector in 36 countries | Structural | Drug availability | Observations * |
| Mendis, 2007 [80] | Bangladesh, Brazil, Malawi, Nepal, Pakistan, Sri Lanka | | 20-50 public (hospital & dispensaries) and 20-60 private for-profit (pharmacies, drug stores) medicine outlets in each of 6 LMIC | Structural | Drug availability | Observations * |
| Muhuri, 1996 [81] | Egypt, Kenya, Tunisia, Uganda, Bolivia, Guatemala | | 95-316 children visiting public and 72-389 children visiting private providers in each of 6 countries | Technical | Clinical practice | Household survey |
| Peabody, 2007 [82] | China, El Salvador, India, Mexico, Philippines | | 63 public and 41 private physicians in 5 LMIC | Technical | Competence | Case scenarios/ vignettes * |

* Comparisons that could be converted to 100% scale and included in analysis

**References of Table S9**

1. Babar ZU, Ibrahim MI, Singh H, Bukahri NI, Creese A (2007) Evaluating drug prices, availability, affordability, and price components: implications for access to drugs in Malaysia. PLoS medicine 4: e82.

2. Barber SL, Gertler PJ, Harimurti P (2007) Differences in access to high-quality outpatient care in Indonesia. Health affairs (Project Hope) 26: w352-366.

3. Garner P, Thomason J, Donaldson D (1990) Quality assessment of health facilities in rural Papua New Guinea. Health Policy and Planning 5: 49-59.

4. Lim MK, Yang H, Zhang T, Feng W, Zhou Z (2004) Public perceptions of private health care in socialist China. Health affairs (Project Hope) 23: 222-234.

5. Liu Y, Berman P, Yip W, Liang H, Meng Q, et al. (2006) Health care in China: the role of non-government providers. Health policy (Amsterdam, Netherlands) 77: 212-220.

6. Meng Q, Liu X, Shi J (2000) Comparing the services and quality of private and public clinics in rural China. Health policy and planning 15: 349-356.

7. Peters DH, Becker S (1991) Quality of care assessment of public and private outpatient clinics in Metro Cebu, The Philippines. International Journal of Health Planning and Management6(4)()(pp 273-286), 1991Date of Publication: 1991: 273-286.

8. Pongsupap Y, Van Lerberghe W (2006) Choosing between public and private or between hospital and primary care: responsiveness, patient-centredness and prescribing patterns in outpatient consultations in Bangkok. Tropical medicine & international health : TM & IH 11: 81-89.

9. Prempree P, Detels R, Ungkasrithongkul M, Meksawasdichai S, Panthong S, et al. (2007) The sources of treatment of sexually transmissible infections in a rural community in central Thailand. Sexual health 4: 17-19.

10. Sihavong A, Lundborg CS, Syhakhang L, Vernby A, Panyanouvong A, et al. (2007) Health providers' competence in the management of reproductive tract infections in Vientiane, Lao People's Democratic Republic. International journal of STD & AIDS 18: 774-781.

11. Syhakhang L, Stenson B, Wahlstrom R, Tomson G (2001) The quality of public and private pharmacy practices. A cross sectional study in the Savannakhet province, Lao PDR. European journal of clinical pharmacology 57: 221-227.

12. Tuan T, Dung VT, Neu I, Dibley MJ (2005) Comparative quality of private and public health services in rural Vietnam. Health policy and planning 20: 319-327.

13. Nordyke RJ (2002) Determinants of PHC productivity and resource utilization: a comparison of public and private physicians in Macedonia. Health policy (Amsterdam, Netherlands) 60: 67-96.

14. Barber SL (2006) Public and private prenatal care providers in urban Mexico: how does their quality compare? International Journal for Quality in Health Care 18: 306-313.

15. Barber SL, Bertozzi SM, Gertler PJ (2007) Variations in prenatal care quality for the rural poor in Mexico. Health Aff (Millwood) 26: w310-323.

16. Bojalil R, Guiscafre H, Espinosa P, Martinez H, Palafox M, et al. (1998) The quality of private and public primary health care management of children with diarrhoea and acute respiratory infections in Tlaxcala, Mexico. Health Policy and Planning 13: 323-331.

17. Lewis M, Eskeland G, Traa-Valerezo X (2004) Primary health care in practice: is it effective? Health policy (Amsterdam, Netherlands) 70: 303-325.

18. Peabody JW, Rahman O, Fox K, Gertler P (1994) Quality of care in public and private primary health care facilities: structural comparisons in Jamaica. Bull Pan Am Health Organ 28: 122-141.

19. Waters HR, Hatt LE, Black RE (2008) The role of private providers in treating child diarrhoea in Latin America. Health Economics 17: 21-29.

20. Abdo-Rabbo A (2003) Prescribing rationality and availability of antimalarial drugs in Hajjah, Yemen. Eastern Mediterranean health journal = La revue de sante de la Mediterranee orientale = al-Majallah al-sihhiyah li-sharq al-mutawassit 9: 607-617.

21. Aounallah Skhiri H, Ben Romdhane H, Chebbi R, Gharbi D, Ennigrou S, et al. (2005) [Management of arterial hypertension: results of a Tunisian general practitioner survey]. La Tunisie medicale 83 Suppl 5: 47-52.

22. Langsten R, Hill K (1995) Treatment of childhood diarrhea in rural Egypt. Soc Sci Med 40: 989-1001.

23. Ministry of Health and Population [Egypt], El-Zanaty Associates, ORC Macro (2003) Egypt Service Provision Assessment Survey 2002. Calverton, Maryland, USA.

24. Ministry of Health and Population, El-Zanaty Associates, ORC Macro (2005) Egypt Service Provision Assessment Survey 2004. Calverton, Maryland, USA.

25. Nikniyaz A, Farahbakhsh M, Ashjaei K, Tabrizi D, Sadeghi-Bazargani H, et al. (2006) Maternity and child health care services delivered by public health centers compared to health cooperatives: Iran's experience. Journal of Medical Sciences6(3)()(pp 352-358), 2006Date of Publication: May 2006: 352-358.

26. Simon N, Hakkou F, Minani M, Jasson M, Diquet B (1998) [Drug prescription and utilization in Morocco]. Therapie 53: 113-120.

27. Akin JS, Hutchinson P (1999) Health-care facility choice and the phenomenon of bypassing. Health policy and planning 14: 135-151.

28. Bhatia JC, Cleland J (2001) Health-care seeking and expenditure by young Indian mothers in the public and private sectors. Health policy and planning 16: 55-61.

29. Das J, Hammer J (2007) Location, location, location: residence, wealth, and the quality of medical care in Delhi, India. Health affairs (Project Hope) 26: w338-351.

30. International Institute for Population Sciences (IIPS), ORC Macro (2000) National Family Health Survey (NFHS-2), 1998/99: India. Mumbai: IIPS.

31. Janjua NZ, Razaq M, Chandir S, Rozi S, Mahmood B (2007) Poor knowledge--predictor of nonadherence to universal precautions for blood borne pathogens at first level care facilities in Pakistan. BMC Infect Dis 7: 81.

32. Kotwani A (2003) Medicine prices in the state of Rajasthan, India: Survey report. New Delhi: Delhi Society for Promotion of Rational Use of Drug. .

33. Kotwani A (2009) Availability, price and affordability of asthma medicines in five Indian states. Int J Tuberc Lung Dis 13: 574-579.

34. Kumar R, Taneja DK, Dabas P, Ingle GK, Saha R (2005) Knowledge about tetanus immunization among doctors in Delhi. Indian journal of medical sciences 59: 3-8.

35. Mahaprata P (2003) Quality Health Care in Private and Public Health Care Institutions In: Abdo S, Yazbeck AS, Peters DH, editors. Health Policy Research in South Asia: Building Capacity for Reform. Washington DC: The World Bank. pp. 333-367.

36. Patel V, Vaidya R, Naik D, Borker P (2005) Irrational drug use in India: a prescription survey from Goa. Journal of postgraduate medicine 51: 9-12.

37. Siddiqi S, Hamid S, Rafique G, Chaudhry SA, Ali N, et al. (2002) Prescription practices of public and private health care providers in Attock District of Pakistan. The International journal of health planning and management 17: 23-40.

38. Vandan N, Ali M, Prasad R, Kuroiwa C (2008) Physicians' knowledge regarding the recommended anti-tuberculosis prescribed medication regimen: a cross-sectional survey from Lucknow, India. Southeast Asian J Trop Med Public Health 39: 1072-1075.

39. Ahmed AM, Urassa DP, Gherardi E, Game NY (1996) Patients' perception of public, voluntary and private dispensaries in rural areas of Tanzania. East African medical journal 73: 370-374.

40. Akin JS, Guilkey DK, Denton EH (1995) Quality of services and demand for health care in Nigeria: a multinomial probit estimation. Social science & medicine (1982) 40: 1527-1537.

41. Akoria OA, Isah AO (2008) Prescription writing in public and private hospitals in Benin City, Nigeria: the effects of an educational intervention. The Canadian journal of clinical pharmacology = Journal canadien de pharmacologie clinique 15: e295-305.

42. Bitran R (1995) Efficiency and quality in the public and private sectors in Senegal. Health policy and planning 10: 271-283.

43. Boller C, Wyss K, Mtasiwa D, Tanner M (2003) Quality and comparison of antenatal care in public and private providers in the United Republic of Tanzania. Bulletin of the World Health Organization 81: 116-122.

44. Cheraghali AM, Idries AM (2009) Availability, affordability, and prescribing pattern of medicines in Sudan. Pharm World Sci 31: 209-215.

45. Commeyras C, Ndo JR, Merabet O, Kone H, Rakotondrabe FP (2006) [Health and drug consumption profile in Cameroon]. Sante (Montrouge, France) 16: 13-19.

46. Fawole AO, Onyeaso NC (2008) Perception and practice of malaria prophylaxis in pregnancy among primary health care providers in Ibadan, Nigeria. West Afr J Med 27: 92-96.

47. Gilson L, Magomi M, Mkangaa E (1995) The structural quality of Tanzanian primary health facilities / L. Gilson, M. Magomi, & E. Mkangaa. Bulletin of the World Health Organization 73: 105-114.

48. Ghana Statistical Service (GSS), Health Research Unit Ministry of Health, ORC Macro (2003) Ghana Service Provision Assessment Survey 2002. Calverton, Maryland.

49. Huebner RE, Moeti TL, Binkin NJ, Rumisha DW (1997) Survey of physician use of radiography and sputum smear microscopy for tuberculosis diagnosis and follow-up in Botswana. The international journal of tuberculosis and lung disease : the official journal of the International Union against Tuberculosis and Lung Disease 1: 333-338.

50. Kanji N, Kilima P, Lorenz N, Garner P (1995) Quality of primary outpatient services in Dar-es-Salaam: a comparison of government and voluntary providers. Health Policy Plan 10: 186-190.

51. Leonard KL, Masatu MC (2007) Variations in the quality of care accessible to rural communities in Tanzania. Health affairs (Project Hope) 26: w380-392.

52. Lindelow M, Reinikka R, Svensson J (2003) Health care on the front lines: Survey evidence on public and private providers in Uganda. 38, , . Washington DC: Human Development Sector, Africa Region, World Bank.

53. Maiga D, Diawara A, Maiga MD (2006) [Evaluation of rational prescribing and dispensing of medicines in Mali]. Rev Epidemiol Sante Publique 54: 497-505.

54. Mariko M (2003) Quality of care and the demand for health services in Bamako, Mali: the specific roles of structural, process, and outcome components. Social science & medicine (1982) 56: 1183-1196.

55. Massele AY, Sayi J, Nsimba SE, Ofori-Adjei D, Laing RO (1993) Knowledge and management of malaria in Dar es Salaam, Tanzania. East African medical journal 70: 639-642.

56. Massele AY, Nsimba SE (1997) Comparison of drug utilisation in public and private primary health care clinics in Tanzania. East African medical journal 74: 420-422.

57. Mbanefoh GF, Soyibo A, Anyanwu JC (2004) Markets for Health Care Nigeria In: Nwabu G, Wang’ombe J, Okello D, Munishi G, editors. Improving Health Policy in Africa. Nairobi: University of Nairobi Press. pp. 431–441.

58. Mliga GR (2003) Decentralization and the quality of health care in Tanzania. In: Leonard DK, editor. Africa's changing markets for health and veterinary services: the new institutional issues. UC Berkeley, GAIA Books, Global, Area, and International Archive: GAIA Books.

59. Ministry of Health [Kenya], National Council for Population and Development [Kenya], ORC Macro (2000) Kenya Service Provision Assessment Survey 1999. Calverton, Maryland.

60. Ministry of Health [Rwanda], National Population Office [Rwanda], ORC Macro (2003) Rwanda Service Provision Assessment Survey 2001. Calverton, Maryland.

61. Ministry of Health and Social Welfare (MOHSW) Tanzania Mainland, MOWSW Zanzibar, World Health Organization (2007) Tanzania Service Availability Mapping 2005-2006. Geneva, Switzerland.

62. Mouyokani J, Tursz A, Crost M, Cook J, Nzingoula S (1999) [An epidemiological study of consultations of children under 5 years of age in Brazzaville (Congo)]. Rev Epidemiol Sante Publique 47 Suppl 2: 2S115-131.

63. Mwabu G, Ainsworth M, Nyamete A (1993) Quality of medical care and choice of medical treatment in Kenya. Journal of Human Resources 28: 838-862.

64. Nahum A, Akogbeto M (2000) [Malaria and pregnancy: attitude of health care personnel during prenatal care in Cotonou, Benin]. Med Trop (Mars) 60: 251-255.

65. National Buerau of Statistics (NBS) [Tanzania], Macro International Inc (2007) Tanzania Service Provision Assessment Survey 2006. . Dar es Salaam, Tanzania.

66. National Coordinating Agency for Population and Development (NCAPD) [Kenya], Ministry of Health (MOH), Central Bureau of Statistics (CBS), ORC Macro (2005) Kenya Service Provision Assessment Survy 2004. Nairobi, Kenya.

67. Nhachi CF, Kasilo OJ (1993) Perception of hospital pharmacists/dispensing personnel of the essential drugs concepts (EDC) in Zimbabwe. East African medical journal 70: 94-97.

68. National Institute of Statistics (NIS) [Rwanda], Ministry of Health (MOH) [Rwanda], Macro International Inc (2008) Rwanda Service Provision Assessment Survey 2007. Calverton, Maryland, USA.

69. Nshuti L, Neuhauser D, Johnson JL, Adatu F, Whalen CC (2001) Public and private providers' quality of care for tuberculosis patients in Kampala, Uganda. The international journal of tuberculosis and lung disease : the official journal of the International Union against Tuberculosis and Lung Disease 5: 1006-1012.

70. Ogwal-Okeng JW, Obua C, Waako P, Aupont O, Ross-Degnan D (2004) A comparison of prescribing practices between public and private sector physicians in Uganda. East African medical journal Suppl: S12-16.

71. Pappas G, Aronovich D, Mukooyo E, Bunnell R, Kabatesi D, et al. (2004) Access to basic HIV/AIDS-related clinical services in Uganda: Results of a nationally representative health facility survey. AIDS and Public Policy Journal19(3-4)()(pp 75-85), 2004Date of Publication: Sep 2004: 75-85.

72. Richard V, Morel B, Wadack NA, Banguio M (2004) [Factors explaining quality of primary health care in Logone Occidental (Chad)]. Rev Epidemiol Sante Publique 52: 249-259.

73. Robertson J, Forte G, Trapsida JM, Hill S (2009) What essential medicines for children are on the shelf? Bulletin of the World Health Organization 87: 231-237.

74. Sondo B, Testa J, Traore C, Soudre R, Tiendrebeogo H (1999) [Capability of health services of the city of Ouagadougou to diagnose sexually transmitted diseases]. Rev Epidemiol Sante Publique 47: 323-328.

75. Suleiman BA, Houssein AI, Mehta F, Hinderaker SG (2003) Do doctors in north-western Somalia follow the national guidelines for tuberculosis management? Eastern Mediterranean health journal = La revue de sante de la Mediterranee orientale = al-Majallah al-sihhiyah li-sharq al-mutawassit 9: 789-795.

76. Tann CJ, Kizza M, Morison L, Mabey D, Muwanga M, et al. (2007) Use of antenatal services and delivery care in Entebbe, Uganda: a community survey. BMC pregnancy and childbirth 7: 23.

77. Voeten HA, Otido JM, O'Hara HB, Kuperus AG, Borsboom GJ, et al. (2001) Quality of sexually transmitted disease case management in Nairobi, Kenya: a comparison among different types of healthcare facilities. Sexually transmitted diseases 28: 633-642.

78. Vuylsteke B, Traore M, Mah-Bi G, Konan Y, Ghys P, et al. (2004) Quality of sexually transmitted infections services for female sex workers in Abidjan, Cote d'Ivoire. Tropical medicine & international health : TM & IH 9: 638-643.

79. Cameron A, Ewen M, Ross-Degnan D, Ball D, Laing R (2009) Medicine prices, availability, and affordability in 36 developing and middle-income countries: a secondary analysis. Lancet 373: 240-249.

80. Mendis S, Fukino K, Cameron A, Laing R, Filipe A, et al. (2007) The availability and affordability of selected essential medicines for chronic diseases in six low- and middle-income countries (Brief record). SO: Bulletin of the World Health Organization 85: 279-288.

81. Muhuri PK, Anker M, Bryce J (1996) Treatment patterns for childhood diarrhoea: evidence from demographic and health surveys. Bulletin of the World Health Organization 74: 135-146.

82. Peabody JW, Liu A (2007) A cross-national comparison of the quality of clinical care using vignettes. Health Policy and Planning22(5)()(pp 294-302), 2007Date of Publication: Sep 2007: 294-302.
